# Neural Responses to Visual Food Cues According to Weight Status: A Systematic Review of Functional Magnetic Resonance Imaging Studies

**DOI:** 10.3389/fnut.2014.00007

**Published:** 2014-07-09

**Authors:** Kirrilly M. Pursey, Peter Stanwell, Robert J. Callister, Katherine Brain, Clare E. Collins, Tracy L. Burrows

**Affiliations:** ^1^School of Health Sciences, Priority Research Centre for Physical Activity and Nutrition, University of Newcastle, Callaghan, NSW, Australia; ^2^School of Health Sciences, Priority Research Centre for Translational Neuroscience and Mental Health, University of Newcastle, Callaghan, NSW, Australia; ^3^School of Biomedical Sciences and Pharmacy, Priority Research Centre for Translational Neuroscience and Mental Health, University of Newcastle, Callaghan, NSW, Australia

**Keywords:** functional magnetic resonance imaging, fMRI, food cues, visual, obesity, weight status

## Abstract

Emerging evidence from recent neuroimaging studies suggests that specific food-related behaviors contribute to the development of obesity. The aim of this review was to report the neural responses to visual food cues, as assessed by functional magnetic resonance imaging (fMRI), in humans of differing weight status. Published studies to 2014 were retrieved and included if they used visual food cues, studied humans >18 years old, reported weight status, and included fMRI outcomes. Sixty studies were identified that investigated the neural responses of healthy weight participants (*n* = 26), healthy weight compared to obese participants (*n* = 17), and weight-loss interventions (*n* = 12). High-calorie food images were used in the majority of studies (*n* = 36), however, image selection justification was only provided in 19 studies. Obese individuals had increased activation of reward-related brain areas including the insula and orbitofrontal cortex in response to visual food cues compared to healthy weight individuals, and this was particularly evident in response to energy dense cues. Additionally, obese individuals were more responsive to food images when satiated. Meta-analysis of changes in neural activation post-weight loss revealed small areas of convergence across studies in brain areas related to emotion, memory, and learning, including the cingulate gyrus, lentiform nucleus, and precuneus. Differential activation patterns to visual food cues were observed between obese, healthy weight, and weight-loss populations. Future studies require standardization of nutrition variables and fMRI outcomes to enable more direct comparisons between studies.

## Introduction

The prevalence of obesity is increasing rapidly (1) with 33.6% of people in the United States classified as overweight and 34.9% classified as obese in 2011–2012 (2). Obesity increases the risk for a variety of lifestyle diseases including cardiovascular disease, hypertension, diabetes, and some cancers (3), as well as reduced quality of life (4). To minimize the substantial economic and health burden of obesity, numerous approaches have been used to target overweight and obese individuals to facilitate weight loss, including lifestyle and surgical interventions (5). Weight-loss interventions focusing on behavioral changes such as dietary modifications and increased physical activity are commonly used; however, these have demonstrated variable effects on weight loss and its long-term maintenance (6). Recently, there has been interest in the possible role that neural mechanisms play in the development and maintenance of obesity. In addition, increasing attention has been given to investigating the impact these neural mechanisms may have on weight loss and maintenance.

It has been suggested that neural responses to specific foods parallel those that are observed in drug dependence and chronic addiction (7, 8). To date, neuroimaging techniques, such as functional magnetic resonance imaging (fMRI), have provided a technique to report the activation of reward-related brain regions in response to food. Visual and olfactory food cues as well as actual food intake have been shown to activate similar brain regions to that of illicit drugs (9–13) in susceptible individuals (14, 15). These fMRI studies provide new insights into the neurobiology of eating behavior and food-cue responsivity, and suggest that abnormal eating behaviors such as overeating in obesity involve alterations in an individual’s neurocircuitry (9, 10). This could have a significant impact on weight status, and potentially contribute to the current prevalence of obesity. Additionally, alterations in neurocircuitry could provide an explanation for some of the lack of effectiveness of weight-loss interventions, as well as maintenance of weight loss in susceptible individuals (16–18).

Many fMRI studies have attempted to identify the neural correlates of eating behavior that could potentially lead to obesity. A great deal of heterogeneity, however, is evident in study design and methodological techniques across the available studies to date (19–21). Previous literature on the neural processing of visual food cues has identified alterations in limbic, paralimbic, and frontal brain circuits. These brain areas are associated with emotional salience, memory, reward, and cognitive and visual processing. Furthermore, motivational state, weight status, and energy density of presented foods have been reported to affect neural responses (20–24). Reviews of the literature investigating neural responses to food cues to date have included individuals with eating disorders or used multiple stimuli modalities such as taste. These approaches may affect neural responses to food cues (20) or recruit multiple anatomical centers in the brain (21, 25).

Specific meta-analysis has emerged as a method to overcome the heterogeneity in fMRI studies. Activation likelihood estimation (ALE) meta-analysis is a technique that integrates findings of fMRI experiments to identify common or divergent activation patterns across a range of studies, using standardized brain coordinates. Existing ALE meta-analyses assessing neural activation of healthy weight individuals to visual food cues have shown that the hunger-state and salience of the presented food items alters activity in brain regions associated with arousal, reward processing, attention, visual processing, and memory of previous food experiences (22). Subsequent meta-analyses have found that overweight and obese individuals have altered activity in brain regions associated with related-cue processing, decision making, anticipation, caloric appraisal, arousal, and memory (23, 24).

Currently, the process by which the human brain integrates food signals to produce maladaptive eating behaviors such as overeating in obesity is largely unknown and warrants further investigation. No studies to date have systematically reviewed the neural responses to visual food cues across all weight categories in individuals who do not have a diagnosed history of abnormal eating behavior such as an eating disorder. Additionally, no studies have systematically reviewed published studies or applied meta-analytical techniques to neural responses to visual food cues pre- and post-weight loss. This is important as it could have implications for the development of more effective weight-loss treatments and maintenance of lost weight.

The aim of this systematic review was to examine published literature related to neural activation, as measured by fMRI, in response to visual food cues by weight status. The primary aim of the review was to determine whether differential neural responses are observed when viewing visual food cues based on body mass index (BMI) category. A secondary aim of the review was to determine whether different neural activation patterns are observed in response to viewing food compared to non-food cues in individuals before and after weight loss.

## Materials and Methods

**Figure 1:**
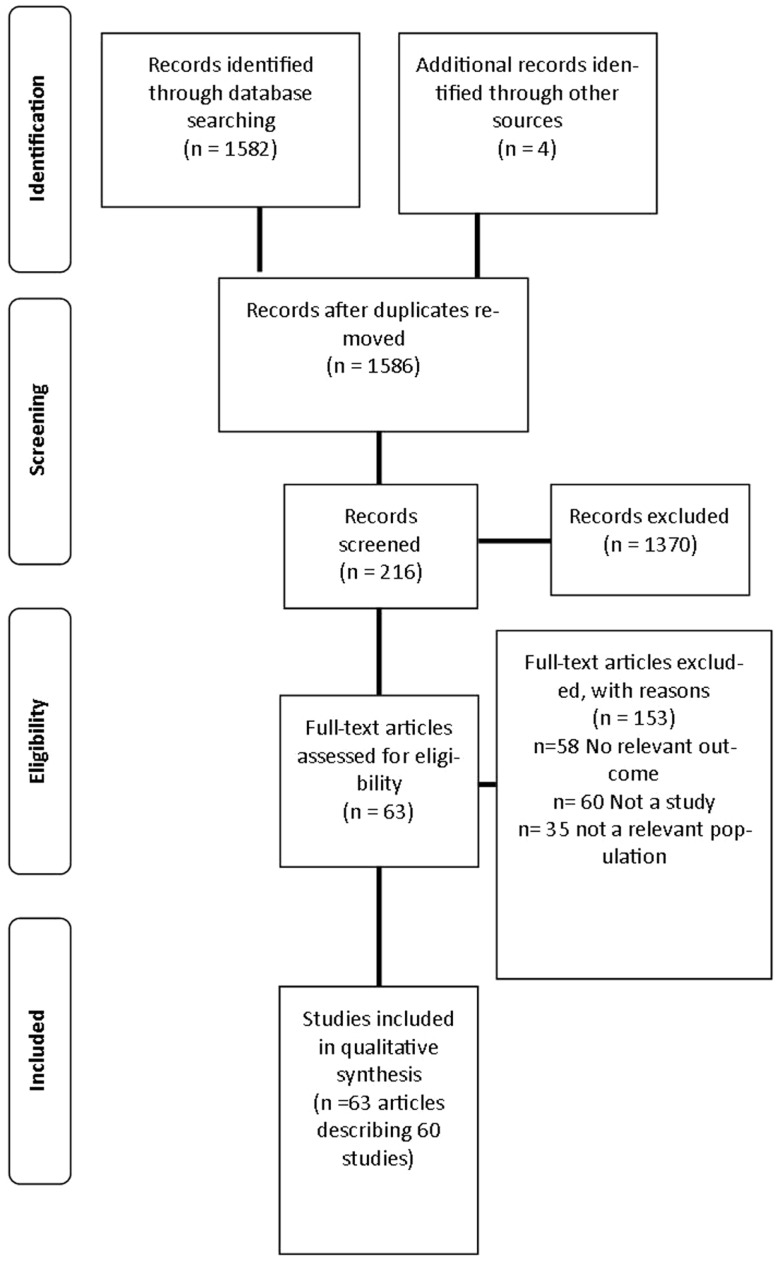
**Flow diagram of studies included in the review**.

A review was undertaken to identify published studies in the English language that used fMRI as a primary outcome measure of neural responses to visual food cues from 1973 to March 2014. This process is outlined in Figure 1.

Initially, electronic databases were searched including: MEDLINE, The Cochrane Library, EMBASE (Excerpta Medica Database), CINAHL (Cumulative Index to Nursing and Allied Health), Informit Health Collection, Proquest, Web of Science, Scopus, and PsycINFO. A pre-determined list of keyword search terms was informed and compiled from a preliminary search of the literature and expanded medical subject headings (MeSH). Keywords were used individually and in combination and included: fMRI, blood oxygen level dependent (BOLD), functional imaging, BOLD signal, BOLD effect, oxyhemoglobin, and deoxyhemoglobin, reward, overeating, addiction, process addiction, food addiction, binge, craving, and dopamine. In addition, electronic searches were supplemented by systematically checking reference lists of relevant publications.

Following the removal of duplicate references, titles and abstracts of identified studies were assessed by two independent reviewers (KP and PS). A predetermined inclusion criterion was applied to determine the study’s eligibility in the review. Studies were included if they investigated an adult population (>18 years of age), used visual food cues, reported weight status, and included fMRI as an outcome measure. Studies reporting a range of BMI categories were included to examine the relationship between neural activation and weight status. Healthy weight individuals were included in the review to act as a comparative group for examining brain activation to visual food cues across all weight status categories. Studies involving participants with a previous or current eating disorder including anorexia nervosa, bulimia nervosa, and binge-eating disorder were excluded, as these participants may have variable responses to food cues that could be attributed to the diagnosis of an eating disorder. Additionally, children or adolescents (<18 years of age); participants with mental and neurological disorders including Prader–Willi Syndrome; pharmacological interventions and fMRI investigations into food intake alone were excluded in keeping with studies included in this review (26–29). If a study included a population meeting the exclusion criteria but reported fMRI outcomes separately for healthy weight and overweight/obese participants, only data on the healthy weight and overweight/obese population was reported in the review. Articles were retrieved for all studies that met the inclusion criteria. If eligibility was unclear, the article was retrieved for further clarification.

Studies were quality checked by two independent reviewers using a standardized 10-question tool (30). The assessed quality criteria included the source of funding, method of sample selection, intervention description, study blinding, and statistical analysis. Four of the quality criteria were designated as “important” and needed to be met to receive a high quality rating. These included: sample selection, comparability of study groups, intervention description, and validity and reliability of outcome measures. An overall classification quality was assigned to each study. Studies were classified as positive quality if >5/10 criteria were satisfied and all important criteria were met. If the majority of criteria were satisfied but at least one of the important criteria was not met, the study was classified as neutral quality. If the majority of the criteria (>5/10) or important questions (≥2/4) were not satisfied, the study was classified as negative quality. Criteria were classified as unclear if the reviewers could not determine whether criteria were met from the detail provided in the published article. Additionally, quality-related fMRI outcomes such as cluster size and volume were extracted and reported in the review. No studies were excluded based on quality ratings. Data were extracted using standardized tables developed for the review. In cases of uncertainty of a study’s inclusion, quality assessment or data extraction were resolved by the consultation of a third independent reviewer until consensus was reached.

Studies were grouped and analyzed by BMI using the World Health Association (WHO) classification, i.e., underweight (<18.49 kg/m^2^), healthy weight (18.00–24.99 kg/m^2^), overweight (25.00–29.99 kg/m^2^), or obese (>30.00 kg/m^2^) (31). Four groups were created for analysis including: (1) studies that compared healthy weight individuals to overweight/obese individuals; (2) studies investigating individuals pre- and post-weight loss; (3) studies of healthy weight individuals only; (4) studies of overweight/obese individuals only. For the purposes of this study, individuals classified as underweight using the WHO cut points were included in the healthy weight category. Additionally, in studies where BMI spanned a number of categories, the mean BMI was used to classify the study into a specific weight category.

### Meta-analysis

To determine the convergence of reported coordinates across studies investigating changes in neural responses pre- and post-weight loss, a meta-analysis was undertaken using the Brainmap GingerALE software^1^. The inclusion criteria for the meta-analysis were identical to the systematic review criteria. In addition, studies were required to report fMRI outcomes of changes in neural activation to visual food cues pre- to post-weight loss (surgical and behavioral) using either Talairach or Montreal Neurological Institute (MNI) coordinates. Only articles reporting whole brain analysis results were included as region of interest analysis is known to inflate activation findings (32). Papers reporting Talairach coordinates were converted to MNI coordinates prior to analysis using the GingerALE software.

Activation likelihood estimation meta-analysis applies a statistical modeling technique (32) that uses reported brain coordinates and adjusts for between-subject and between-template variance to generate a 3-dimensional Gaussian kernel. Subsequently, a modeled activation (MA) map is created and individual maps are combined to generate an experimental ALE map. The experimental map is tested against an ALE null distribution map, representing the null hypothesis that there is random variation between activation across the meta-analyzed studies, when the within-study variation remains fixed. A random-effects model is applied, which assumes convergence between different studies that is above chance.

A statistical threshold of *P* < 0.05 False Discovery Rate (FDR), corrected for multiple comparisons and a minimum cluster size of 100 mm^3^ was set. This is consistent with previous meta-analyses in this area to control for publication bias with respect to the reporting of foci (22–24). Results of meta-analyses are presented using the Mango software package^2^.

## Results

The search strategy identified 1586 articles, 216 articles were screened for inclusion with 64 articles describing 60 studies included in the final analysis (26–29, 33–92) as described in Table S1 in Supplementary Material. The primary reasons for exclusion were: the article did not meet inclusion criteria for study design (*n* = 60); no relevant outcome was studied (*n* = 58); and the study investigated a population not specified in the inclusion criteria (*n* = 35). Three additional studies were excluded as they did not report BMI or weight status of participants (93–95).

A total of 1565 participants were included across the studies (mean 26, range 5–100). Age ranged from 18–66 years with the most commonly studied age group being 18–35 years olds (*n* = 42 studies) (26, 28, 29, 33–43, 45–47, 60–66, 68–72, 74–86, 89, 91, 92). Participants were predominantly female and right handed with 26 of the studies exclusively recruiting females (26, 29, 33, 43, 45–48, 56–59, 65, 66, 70–72, 75, 76, 78–81, 83–85, 90–92). The majority of studies were published post 2009 (*n* = 53, 83%) and used a within participants cross-over design (*n* = 25, 42%). No randomized control trials were retrieved by the search criteria.

Seventeen of the included studies compared both obese and healthy weight participants in the same study (26–28, 33–47). Twelve reported outcomes from pre- to post-weight loss (48–59). Methods of weight loss included bariatric surgery (*n* = 7) (48, 49, 52, 53, 57–59) and behavioral nutrition and lifestyle interventions (*n* = 5) (51, 53–56) with follow up in these studies ranging from one month to twelve months. Weight loss ranged from 3.4–25% of original body mass in these studies. Five of the included studies were exclusively conducted in overweight or obese participants (BMI ≥ 25.00 kg/m^2^) (88–92) and 26 studied individuals with a mean BMI in the healthy weight range (BMI < 25.00 kg/m^2^) (29, 60–87). However, eight of the healthy weight studies included participants with BMI’s spanning from the underweight category to the overweight/obese category (29, 67, 69, 74, 78, 79, 81, 82).

As outlined in Table S1 in Supplementary Material, 30 studies (50%) used participants who were fasted prior to fMRI scans (range 2–24 h) (26–29, 40–42, 44–46, 50, 51, 54, 55, 64, 67, 73, 75, 77–82, 86, 87, 90–92). Seven studied satiated participants (43, 47, 48, 58–60, 76, 83–85) and 23 investigated neural responses in both fasted and satiated conditions (27, 33–39, 52, 53, 56, 57, 61–63, 65, 66, 68–72, 74, 88, 89). The most common variables other than fMRI assessed in the included studies were hunger (*n* = 34 studies), appetite (*n* = 10 studies), and liking ratings of presented foods (*n* = 10 studies).

Food images used in the studies were described by their authors as “high-calorie” foods in 36 of the 60 studies (26, 28, 29, 35, 38, 40, 41, 43–50, 54–61, 64–66, 68, 69, 71, 76, 78–81, 83–88, 90–92) and included foods such as chocolate, chips, and hamburgers. Foods described as “low-calorie” foods were used in 32 studies (28, 35, 38, 40, 42, 44, 46–48, 50, 54–61, 64–66, 68, 69, 71, 78–81, 83–88, 90–92) and included foods such as fruit and vegetables. The actual calorific values for foods were only reported in seven of the studies (35, 47, 57–59, 78, 88). No studies reported the use of a dietitian or nutritionist in the selection or classification of foods. Fifteen studies used foods based on the appeal and salience of the food (e.g., “hedonic,” “palatable,” and “appetizing”) (27, 33, 34, 41, 43, 52, 62, 63, 70, 73, 75, 77–79, 81, 82). Food images were selected using pilot ratings of palatability, perceived calorific value, and recognizability of presented images in only 19 studies (27, 36, 37, 40, 43, 44, 52, 53, 61, 68, 70, 71, 74, 77, 79–82, 86). Control images were used in the majority (*n* = 48) of the included studies and varied greatly, including images of cars, office equipment, landscapes, and blurred images.

Block design was used in 38 of the studies (27, 28, 34, 35, 37–42, 44, 46–53, 55, 56, 60–64, 67–69, 71–73, 81–85, 87–89, 91, 92) and a 3 T magnet was used most commonly to acquire imaging data (*n* = 43) (26, 28, 29, 34, 36, 37, 39–46, 49–53, 55, 56, 60–63, 65–69, 71–73, 75, 76, 78–82, 87, 89–92) (Table S2 in Supplementary Material). The imaging plane most commonly used to acquire images of the brain was the transverse plane parallel to the anterior commissure posterior commissure line (AC–PC line) (*n* = 18).The method of reporting fMRI results was variable across the range of studies. All studies excluding two reported Talairach or MNI coordinates, but only 32 (53%) studies reported cluster size or volume of activation (35, 38, 39, 41, 43, 44, 46, 49, 50, 52, 53, 55–59, 62–64, 66–69, 72, 74, 77, 78, 81, 84–86, 88, 91).

### Healthy weight compared to overweight/obese participants

Across studies comparing overweight and obese participants to healthy weight controls, overweight/obese individuals had greater brain activity to foods compared to non-foods in areas associated with a variety of functions in the context of food-cue processing (96). This included areas associated with reward processing [insula (26, 33, 41, 43, 47), orbitofrontal cortex (OFC) (26, 28, 43)], reinforcement and adaptive learning [amygdala (27, 28, 33, 43), putamen (28, 41, 47), OFC (26, 28, 43)], emotional processing [insula (26, 33, 41, 43, 47), amygdala (27, 28, 33, 43), cingulate gyrus (44, 45)], recollective, and working memory [amygdala (27, 28, 33, 43), hippocampus (27, 28, 33, 45), thalamus (33, 41), posterior cingulate cortex (27, 47), caudate (28, 45, 47)], executive functioning [prefrontal cortex (PFC) (28, 43), caudate (28, 45, 47), cingulate gyrus (44, 45)], decision making [OFC (26, 28, 43), PFC (28, 43), thalamus (33, 41)], and visual processing [thalamus (33, 41), fusiform gyrus (27, 43)]. Additionally, obese individuals displayed greater activation to food cues in areas involved in motor learning and coordination such as hand-to-mouth movements and swallowing [insula (26, 33, 41, 43, 47), putamen (28, 41, 47), thalamus (33, 41), caudate (28, 45, 47)] as well as risk aversion [inferior frontal gyrus (41, 44)]. These increases in brain activity were particularly evident in response to high-calorie foods compared to low-calorie foods. When satiated, increased activity was observed in obese compared to healthy weight individuals in areas involved in decision making [PFC (34, 39), OFC, and caudate (38)], reward anticipation [anterior cingulate (33, 38) and OFC (33, 38)] as well as emotional processing [insula (33, 37), caudate (38), and amygdala (33)]. Significant correlations between BMI and activation were reported in three studies (37, 44, 47).

### Weight change interventions

In three studies using a nutrition and lifestyle intervention, brain activation at the commencement of the intervention was associated with degree of weight-loss success and maintenance. This included areas associated with reward processing and anticipation [insula, anterior cingulate cortex, nucleus accumbens (55), and the OFC (56)], decision making [PFC (54) and OFC (56)], and impulsivity [nucleus accumbens and the anterior cingulate cortex (55)]. Participants who had successfully lost and maintained weight-loss displayed differential neural responses to food cues to those of healthy weight participants in areas involved in emotion, memory, and visual processing [cingulate gyrus, parietal cortex (51)], and to that of obese participants in regions associated with emotion, impulse control, and reward-based learning [PFC and the anterior cingulate (50)].

In studies reporting pre- to post-bariatric surgery outcomes, reductions in activity were reported in the insula and putamen. These areas are implicated in interoceptive processing (52, 58) and reinforcement learning (57, 59), respectively. Further, activation of the hypothalamus, which regulates hunger and subsequent food intake, following gastric bypass surgery resembled the responses of healthy weight individuals more closely than responses of obese individuals. More successful weight loss in gastric bypass surgery was associated with increased baseline neural activity of the dorsolateral PFC (49) and unique changes in activity were found depending on the method of weight loss (i.e., behavioral or surgical) (53). A relationship between BMI and activation of areas involved in reward anticipation and impulsivity [anterior cingulate cortex (49) and middle frontal gyrus (52)] was identified in two studies.

### Healthy weight participants

The most common finding across studies of healthy weight participants was that motivational state (i.e., fasted or satiated state) affected brain activation to food. Fasting often increased responses to high-calorie foods in areas associated with processing of reward and stimuli salience [OFC (66, 69, 71, 72), striatum (65, 69, 72), insula (69, 71)], decision making [OFC (66, 69, 71, 72), striatum (65, 69, 72)], implicit learning [OFC (66, 69, 71, 72), putamen (66, 71)], and the processing of visual cues [fusiform gyrus (68, 71, 74)]. Gender differences were identified in responses to food cues with females displaying greater activation in a variety of brain regions implicated in attention, emotion, recollective memory, and decision making (67, 68, 74).

### Obese participants

In obese participants, food compared to non-food images activated areas including the PFC, insula, amygdala, nucleus accumbens (91, 92), and cerebellum (89). These areas are associated with numerous roles which could affect food cue processing including executive functioning, reward processing, and anticipation, reinforcement learning, memory modulation, and motor control. Females showed greater activation in the caudate and OFC when fasted and greater activation in the anterior cingulate cortex when satiated (88). Abdominal adiposity predicted brain activity in one study (92).

### Results of the ALE meta-analysis

**Figure 2:**
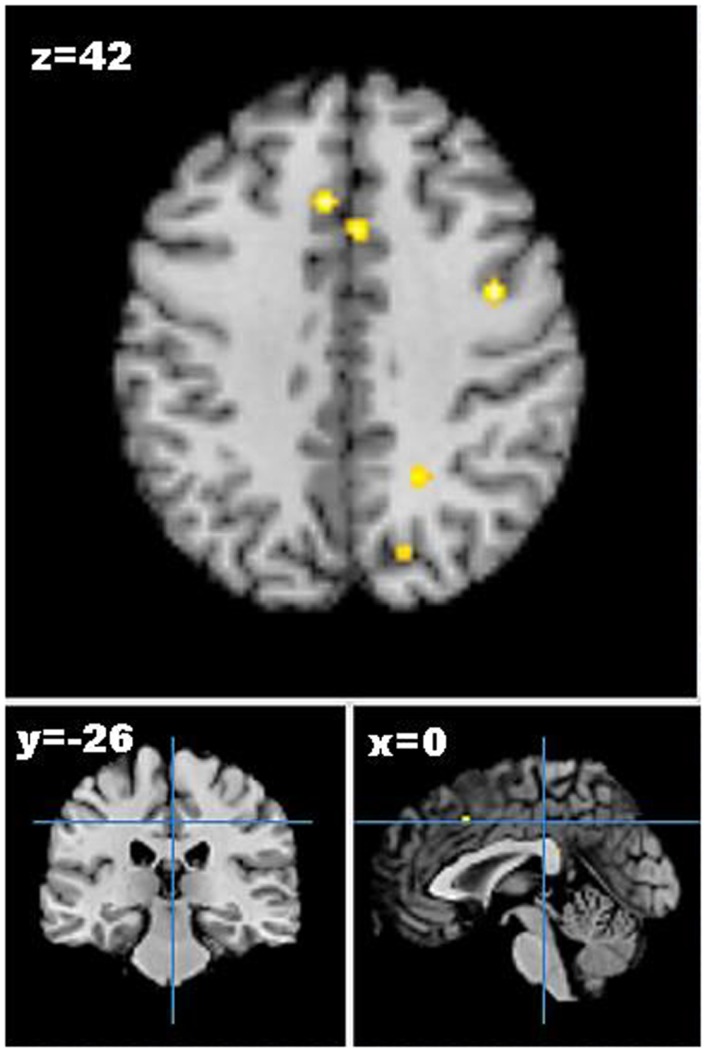
**Axial (*z*), coronal (*y*), and sagittal (*x*) views of decreased activation in studies comparing neural activation to visual food cues from pre- to post-weight loss, as detected by fMRI**. FDR corrected *P * < 0.05, cluster size >100 mm^3^, region of interest studies excluded. Figure shows decreased activation from pre- to post-weight loss in the cingulate gyrus, middle frontal gyrus, and precuneus.

As only one study reported increases in brain activation following weight loss, only studies reporting decreases in neural activation from pre- to post-weight loss were included in the meta-analysis. Five studies describing seven experiments were identified that met the meta-analysis inclusion criteria with 45 participants and 41 foci (55–59). The meta-analysis identified 13 clusters, which survived statistical thresholds, as demonstrated in Figure 2. The largest cluster was the left superior temporal gyrus (MNI: −40, −48, 6), as described in Table S3 in Supplementary Material. Other clusters surviving statistical thresholds included right middle frontal gyrus (MNI: 32, 34, 34), left lentiform nucleus (MNI: −12, 0, −2), left cingulate gyrus (MNI: −4, −34, 26,), and right precentral gyrus (MNI: 40, 0, 42).

## Discussion

This is the first systematic review to investigate neural responses to visual food cues across all weight categories and provide a meta-analysis of neural changes before and after weight loss. This review highlights that neural responses to visual food cues differ depending on the weight status of individuals, and changes in neural activation patterns are observed pre- to post-weight loss. More specifically, obese individuals compared to healthy weight had increased activation to foods compared to non-foods, and this was most pronounced in response to energy dense foods. This was evident in brain regions associated with the anticipation of the rewarding value of the food, emotion and memory associated with previous experiences with the food, and visual processing of the food cues. Obese individuals were more responsive to food cues in the satiated state compared to healthy weight individuals. Additionally, weight loss reduced neural responses in areas related to executive functioning, impulsivity, and reinforcement learning to visual food cues, despite differences in the modality of the intervention. However, inconsistent activation patterns were reported across studies, which may be attributable to the variety of participant groups recruited, pre-scan preparation, and the chosen fMRI parameters (e.g., block design vs. event-related). The fMRI findings of the current review are consistent with previous published reviews in the area (19–21).

When comparing healthy weight and overweight/obese participants, increased reward-related responses to food (e.g., insula and OFC), particularly high-calorie foods, compared to non-food were found in obese participants. It has been suggested that obesity may be linked to an increase in neural-related reward anticipation from food cues, and a decrease in reward during food consumption. This could potentiate overeating to compensate for imbalances in the neural reward pathways and subsequent diminished experience of reward (7, 97). Although only three studies reported a correlation between neural responses and BMI (37, 44, 47), the increased responses reported in these studies of obese participants compared to normal-weight participants may explain some individual’s vulnerability to overeating, food cues, and possible diet failures (7, 10). Similar findings have been reported by Garcia-Garcia et al. and Asmaro et al. who noted differential activation patterns between obese and healthy weight individuals to the sight of food, particularly in reward-related regions. However, individuals with binge-eating disorder and studies using taste stimuli were also included in previous reviews, which may have recruited additional areas of the brain and potentially confound the findings of these studies.

This review extends current literature regarding neural responses to visual food cues by examining brain activation by weight status category (i.e., healthy weight, overweight/obese) as well as pre- and post-weight-loss responses. Results from weight loss studies suggest that gastric bypass surgery reduces reward responses from pre- to post-surgery (49, 52, 57–59). Although consistent reductions in neural activation were observed irrespective of method of weight loss, changes in activation via surgical weight loss differed to activation changes observed in behavioral weight loss that focused on diet and exercise (53). Interestingly, in both behavioral and surgical interventions, brain activity prior to weight loss in areas related to reward anticipation and impulsivity (e.g., anterior cingulate cortex and nucleus accumbens), and decision making (dorsolateral PFC) predicted degree of weight loss success. This provides insight that both surgical and behavioral weight loss may be underpinned by a neural mechanism as well as restriction of the amount of food consumed. In addition, individuals who had maintained successful weight loss showed increased neural activity in regions associated with inhibitory control compared to obese individuals and increased responses in areas related to memory compared to healthy weight individuals (50, 51, 55, 56). The findings of the review highlight that neural-related mechanisms may make some people more predisposed to weight regain, despite successful loss of weight. This may have important implications for obesity follow up and treatment, and provides evidence that neural mechanisms may affect weight loss success or predict proneness to relapse. The high cost of MRI precludes large scale scanning of subjects engaged in weight-loss programs, but use of fMRI in focused clinical trials could be used to validate changes in neurocircuitry patterns associated with successful maintenance of weight loss.

The results of the meta-analysis revealed that there were some small regions of convergence of brain responsivity across weight-loss interventions. Deactivation was observed from pre- to post-weight loss in areas involved in emotion and memory (e.g., cingulate gyrus and precuneus), visual processing (e.g., superior occipital gyrus), learning centers (e.g., lentiform nucleus and cingulate gyrus), and motor regions (e.g., precentral gyrus and lentiform nucleus) (96). This may imply that individuals who have experienced weight loss regardless of modality also have changes in neural activation associated with memory and emotion of previous experiences with the food as well as alterations in the processing of external food inputs. This also suggests that individuals who have experienced weight loss have corresponding changes in activation based on behaviors that are implicitly learned or reinforced during weight-loss interventions. Additionally, it appears that weight loss could result in changes in the planning and regulation of movements associated with eating such as reaching to obtain the food, chewing, and swallowing. Although several areas survived statistical thresholds, volume of activation was small. This suggests that while there were commonalities across studies regarding reductions in neural activity to food cues across weight-loss interventions, the areas of congruence are minimal. This is likely due to the overall number of studies included and the pooling of different modalities of weight loss into a single meta-analysis and thus results should be interpreted accordingly.

Significant differences were found depending on the participant’s motivational state across all weight categories, although fasting times were inconsistent across studies. Obese individuals were found to have activation consistent with continued reward processing (e.g., PFC, OFC, caudate) and emotional responses (e.g., insula, caudate, amygdala) to food cues following a meal compared to healthy weight controls. That is, obese individuals appear to be more reactive to high-calorie foods and have continued reward processing even following a meal. These findings are consistent with previous literature in this area (21, 23, 24). This is significant as it could provide a neural mechanism for overeating in obese individuals, with neural stimulation even in the state of satiety. In the fasting state, obese individuals were found to have increased activation in areas associated with the anticipation of reward while healthy weight controls were found to have greater activation in areas associated with cognitive control. This suggests that BMI and the hunger-state will greatly affect an individual’s natural desire for food and food reward responsiveness, as well as food choices and subsequent caloric intake (14). This provides preliminary data that could be used in weight interventions regarding meal timing to avoid excessive anticipation for food and subsequent overeating. In addition, notable gender bias was found in the current review, with females displaying differential activation patterns compared to males in regions associated with reward anticipation, food motivation, and inhibitory control based on motivational state, i.e., fasted or satiated. (67, 68, 74, 88). This may indicate that gender influences an individual’s susceptibility to addictive-like eating behaviors.

The term ‘food addiction’ has emerged and is being used increasingly in lay literature (98, 99) in association with specific eating patterns and rising levels of obesity (22, 30, 77, 96, 100). It has been postulated that food addiction is associated with specific food-related behaviors including: tolerance to large amounts of food, persistent desire, or craving for specific foods and lack of control over the amount of food consumed (77). In this way food addiction would share similar clinical characteristics that overlap with drug dependence and other common types of addiction, as defined by the Diagnostic and Statistical manual of Mental Disorders version 5 (DSM-V) (29, 77, 101–103). Although, there is debate regarding to the inclusion of food addiction as a DSM-V eating and feeding disorder (3), currently no universally accepted definition for food addiction exists. Findings from this review provide preliminary support for the food addiction hypothesis. Areas of the reward network including the mesolimbic and nigrostriatal regions, which have been implicated in other addictions including drug and alcohol abuse, were activated in response to visual food cues. However, there is a great deal of variation in the brain areas activated across reviewed studies, as well as the experimental conditions used (e.g., motivational state, images presented, fMRI parameters). This indicates that further research utilizing a standardized approach is required to substantiate study findings to either support or refute the existence of food addiction as a distinct phenomenon. This is in line with the findings of Ziauddeen et al. (19) who concluded that while neural activation in healthy weight individuals is somewhat consistent across studies, activation in obese, and binge eaters is too varied to categorically confirm the existence of food addiction.

Although little evidence exists to confirm the types of food associated with food addiction, it is commonly assumed that “junk foods” high in calories, sugar, and fat such as chocolate and potato chips are typical foci of addictive-like tendencies such as craving (27, 76, 104). The range of classifications given to food images in this review such as “high” and “low” calorie foods and “palatable” and “bland” foods appears subjective and arbitrary, with no studies consulting a dietitian or nutritionist. Only seven studies specifically identified actual calorific values of foods and the majority of studies did not pilot test images or provide rationale for their selection of presented foods. The selection of foods in research studies is important to ascertain if it is a universal chemical component of food (e.g., selection based sugar or fat content), a whole category of food (e.g., selection of ‘junk food’), or more simply a personal preference (e.g., selection by administering a pre-scan survey) that could make a food more likely to be overeaten. This could provide further insight into whether food addiction is a plausible phenomenon. Furthermore, the range of control images presented were variable and included cars, landscapes, and blurred images. As these images were not standardized across studies, it is unknown as to whether these assorted images may elicit different neural patterns from one another based on their perceived valence and arousal. The variability of food images used in studies has been previously acknowledged and a database of standardized pictures based on image characteristics and nutrient composition has recently been developed (105). Future studies should consider the use of such a database to facilitate the standardization of images and comparability across studies.

The design of an experimental paradigm for fMRI studies requires extensive planning including behavioral predictions of cognitive tasks and the formulation of a hypothesis to inform the task conditions and image acquisition parameters. Ideally, fMRI experiments cover the whole brain with the highest spatial resolution achievable in the shortest time. The trans-axial plane is usually chosen to cover the whole brain in as few slices as possible. In some cases, the acquisition plane is angled to avoid regions of high magnetic susceptibility (e.g., air-tissue and bone-tissue interfaces) that can adversely affect image quality, such as in targeting the reward network. The block design has dominated fMRI due to its ease of implementation, robustness of results, increased statistical power, and relatively large BOLD signal relative to baseline (106, 107). However, blocked paradigms have poorer temporal resolution and are susceptible to stimulus correlated movement artifacts. Event-related paradigms measure responses to single events (typically over 0.5 s), and then combine a large number of those events to improve statistical power. This is advantageous to detect transient variations in hemodynamic responses, reduce the subject’s ability to predict the next event, and allows for *post hoc* sorting of trials and correlations with other variables (106, 107). Generally, blocked paradigms are most useful to localize activation in brain regions associated with a particular task while event-related paradigms allow for a more in depth investigation of the response profile in an identified brain region.

Across the reviewed studies, fMRI results were not reported in a consistent method. Talairach or MNI coordinates, used to describe the location of brain structures independent from individual differences in the size and overall shape of the brain, were reported in the majority of studies. Using the standardized coordinates allows the comparison of brain region activation with other studies, increasing the power of results when combined and can provide a method for meta-analysis (25). Cluster size or volume indicates that the area of neural activation reported in the study is large enough to be statistically plausible rather than just an error in measurement, improving the quality of the study (108). Only half of the studies included in the review reported cluster size or volume.

This review is limited by the heterogeneity of study variables used across the reviewed articles, making direct comparisons between studies difficult. As a limited number of studies met the inclusion criteria for the meta-analysis comparing pre- and post-weight loss activation changes, a single exploratory meta-analysis was undertaken combining both surgical and behavioral weight-loss interventions. The pooling of different methods of weight loss could impact the findings of the meta-analysis. However, this data provides insight into neural activation following weight loss, regardless of modality, which needs to be substantiated in future research when more studies have been published in this area. The quality of reporting BMI was inconsistent across studies, with participant groups containing BMI ranges that corresponded with more than one of the WHO categories (54). This could affect possible relationships between fMRI outcomes and weight status. As BMI can be affected by numerous factors including muscle mass, adiposity or body fat percentage is a more reflective measure of obesity assessment and should be considered for use in future studies. The majority of reviewed articles studied adult female participants exclusively, potentially limiting the generalizability of the study findings to other population groups. Finally, the broad age range of the study participants could be a potential limitation of the review, with previous studies showing age-related changes in neural activity including reduced sensitivity of brain areas associated with satiety (109).

Strengths of this review include the standardization of populations studied and stimuli used to visual food cues exclusively. The use of different stimuli modalities including food consumption (104, 110–122), odors (123–125), and intravenous infusion (126) recruits additional areas of the brain including taste, texture, olfactory, and food intake centers, potentially confounding results. Only visual food cues have been included in the current review in order to minimize the activation of additional areas of the brain. Additionally, the exclusion of different populations, including children and adolescents (127, 128), eating disorders including binge-eating disorder, anorexia nervosa and bulimia nervosa (129–134), and neurological disorders (39, 135), reduces the chance of additional confounding factors contributing to the variations in neural responses to visual food cues. This exclusion criterion is consistent with previous literature in the field (28, 38, 43).

It is recommended that future studies report detailed nutritional information of images presented during scanning and BMI classification using the WHO guidelines. This field of research requires the involvement of a multidisciplinary team including imaging specialists, neuroscientists, psychologists, and dietitians to ensure high quality study design. Further, studies should use validated eating behavior questionnaires and use within participant cross-over study design to investigate the impact of motivational state on neural responses. Weight-loss studies using fMRI as an outcome measure should routinely report brain coordinates, cluster size and threshold. Future meta-analyses in this area should investigate responsivity to food cues by specific mode of weight loss (i.e., surgical or behavioral) as these are likely to elicit different changes in neural responses.

## Conclusion

This review found that neural activation differed based on weight status with obese individuals displaying increased responses to food compared to non-food and continued responsivity to food following a meal. This suggests that neural activity to food cues could be an additional mechanism contributing to the pathogenesis of overeating and subsequent weight gain. Regions of activation differed across the reviewed studies due to a wide range of study conditions used and inconsistency in reporting of findings. The meta-analysis undertaken revealed changes in brain activation patterns following weight loss. However, the small cluster sizes suggest that there is minimal congruence of neural activation across weight-loss studies. Future fMRI studies examining neural activation in response to visual food cues should standardize reporting of nutrition variables and fMRI outcomes to allow for more direct comparisons between studies.

## Conflict of Interest Statement

The authors declare that the research was conducted in the absence of any commercial or financial relationships that could be construed as a potential conflict of interest.

## Supplementary Material

The Supplementary Material for this article can be found online at http://journal.frontiersin.org/Journal/10.3389/fnut.2014.00007/abstract

## Footnotes

^1^http://www.brainmap.org/index.html

^2^http://rii.uthscsa.edu/mango/
