# Supplementary material for: Neural Responses to Visual Food Cues According to Weight Status: A Systematic Review of Functional Magnetic Resonance Imaging Studies
Source: Front Nutr. 2014 Jul 9;1:7. doi: 10.3389/fnut.2014.00007 (PMC4428493; doi:10.3389/fnut.2014.00007)
Supplement: Supplementary file 1 [file Data_Sheet_1.DOCX]

**Table 1:** Characteristics of included studies investigating neural responses to visual food cues by weight status

| **Author** | **Study design** | **Sample size** | **Population characteristics** | **Study Paradigm** | | **Nutrition standardisation** | **Visual cue details ^a^**  **Task Control** | |
| --- | --- | --- | --- | --- | --- | --- | --- | --- |
| **Healthy weight vs overweight/obese** | | | | | | | | |
| **Connolly 2013 ^33^** | Double blind crossover design | n=22:  2 excluded i.e. n=20, n= 10 HW  n= 10 OB | Female  Age HW: 24.60±1.33yrs, OB 26.50±1.64rs  BMI: HW 22.40±0.50 kg/m^2^, OB 32.91±0.74 kg/m^2^  Right handed, matched for menstrual cycle | | Event-related | 6h fasting.  Scan 1: non-nutritive cranberry beverage  Scan 2: cranberry juice with 300kcal | Appetising food images | Images of brick walls |
| **Cornier 2013 ^34^** | Within participants crossover design | n=56  3 excluded i.e. n=53  Obese Resistant (OR) n=25  Obese Prone (OP) n=28 | OR: female n=11, male= 14, OP: female n=14, male n=14.  Age: OR: 31.4±3.4yrs, OP: 30.2±3.8yrs.  BMI: OR: 20.8±2.0 kg/m^2^, OP: 26.2±2.9 kg/m^2^ (P<0.001).  Right handed, matched for menstrual phase | | Block design | Run in diet eucaloric for 4 days, compliance monitored  Scan 1 (Fasted): Overnight fast >10h  Scan 2 (Satiated): meal 25% energy requirements | Foods of high hedonic value  Foods of low hedonic value | Non-food item |
| **Ho 2012** ^35^ | Within participants crossover design | n=35-  OW/OB n=21  HW n=14 | OW/OB: female n=11, male n=10, HW: female n= 8, male n=6  Age: OW/OB 24.4±6.4yrs, HW 24.5±4.5yrs.  BMI: OW/OB: 30.64±4.1 kg/m^2^, HW 21.83±1.4 kg/m^2^.  Right handed, healthy. | | Block design | Scan 1 (Fasted): 4 h fast  Scan 2 (satiated): 750kcal standardised meal 30mins prior to scan. | High cal foods (200kcal/serve), Low cal (50kcal/serve) | Furniture |
| **Lee 2013 ^36^** | Within participants crossover design | n=30  Low disinhibition (LD) n=14  High disinhibition (HD) n=16 | Male  Age: LD: 28.2±6.24yrs, HD: 25.9±4.61yrs,  BMI: LD: 21.9±2.13 kg/m^2^, HD: 26.8±4.78 kg/m^2^ (P<0.002)  Recruited from larger study, weight stable, right handed | | Event-related | Meal providing 25% of daily energy requirements.  Scan 1 (Fasted): 7h fast following meal  Scan 2 (satiated): second meal providing 20% requirements 45 min prior to scan | Identifiable food images |  |
| **Martens 2013 ^37^** | Within participants crossover design | n=40,  HW n=20  OW/OB n=20 | 45 screened, healthy.  HW female n=10, male n=10, OW/OB: female n=10, male n=10  Age: HW: 22.4±0.5yrs, OW: 23.7±1.0yrs  BMI: HW: 22.7±0.3 kg/m^2^ (range 20-25 kg/m^2^), OW: 28.1±0.5 kg/m^2^ (range 25-35 kg/m^2^) (P<0.0001) | | Block design | Scan 1 (Fasted): overnight fast 10h excluding water.  Scan 2 (Satiated): meal providing 20% daily energy requirements 20mins prior to scan | Food | Non-food |
| **Dimitropoulos 2012^38^** | Within participants crossover design | N= 38  OB n=22  HW n=16 | HW female n=10, male n=6, OB female n=11, male n=11  Age: HW=24.6±4.2yrs, OB=24.8±6.7yrs  BMI: HW: 22.7±1.4 kg/m^2^, OB: 31.6±4.5 kg/m^2^ (P<0.001)  % right handed: HW=94, OB=82 | | Block design | Scan 1 (fasted): light breakfast before 8am; fasting following breakfast  Scan 2: meal prior to scan (750kcal) | High calorie foods  Low calorie foods | Non-food images |
| **Holsen 2012^39^** | Within participants crossover design | n=43.  1 OB excluded  14 OB,  15 HW controls.^b^ | OB female n=9, male n=5, HW: female n=9, male n=6  Age: OB 25.0±10.3, HW 23.1±9.7  HW significantly lower BMI (21.1±2.8 kg/m^2^) than OB (32.4±3.5 kg/m^2^, p<0.01). | | Block design | Fasting condition: fasting 4h  Satiated condition: 500kcal meal 15 mins prior to scan | Food images | Blurred baseline images. Animal images. |
| **Garcia Garcia 2013** ^40^ | Case control | n=37  OB n=18  HW n=19 | OB: female n=13, male n=5, HW: female n=11, male n=8.  Age: OB 34.78±4.45yrs (range 22-39yrs), HW 32.00±5.87yrs (range 21-40yrs).  BMI: OB 34.89±4.78 kg/m^2^ (range 30.1-48.58 kg/m^2^), HW 22.44±1.93 kg/m^2^ (range 19.53-24.97 kg/m^2^).  Menstrual phase controlled | | Block design | Fasting 3-5h | High calorie food (salty and sweet)  Low calorie food | Neutral and Rewarding non-food stimuli |
| **Jastreboff 2013** ^41^ | Case control | N=50  OB n=25  HW n=25 | Female n=19, male n=31  Mean age 26yrs.  BMI OB 32.6±2.2 kg/m^2^, HW 22.9±1.5 kg/m^2^. | | Block design | Fasting 2h | Favourite food | Stress cues, neutral relaxing cues |
| **Kullmann 2013 ^42^** | Case control | n=24,  HW n=12  OW n=3  OB n=9 | HW: female n=6, male n=6, OW/OB: 6 female, 6 male.  Age: HW: 22.91±2.10yrs (range 22-29yrs), OW/OB: 24.66±2.42yrs (range 21-28yrs)  BMI: HW: 21.16±1.13 (kg/m^2^ range 19.4-22.5 kg/m^2^), OW/OB: 30.46± 1.77kg/m^2^ (range 28.4-34.4 kg/m^2^) | | Block design | Overnight fast >10h | High calorie foods  Low calorie foods | Non-foods |
| **Frankort 2012^43^** | Case control | n=28. 1 OW excluded  Overweight n=13 (3 OB)  HW n=15. | Female  Age: HW=23.1±5.4 yrs, O/W=23.9±7.6.  BMI: O/W=29.8±3.3 kg/m^2^ (range >27-<38), HW=21.1±1.1 kg/m^2^ (range >19-<23)  Matched for phase of menstrual cycle | | Event-related | Regular lunch 1-1.5h prior to scan. | Food images: high and low calorie ‘palatable’ and ‘distasteful’ foods |  |
| **Grosshans 2012**^44^ | Case control | N= 44  OB n=21  HW n=23 | OB: Female n=15, male n=6. HW: Female n=15, male n= 8  Age: OB=44.0±12.7yrs, HW=37.7±11.4yrs  BMI: OB=36.9 kg/m^2^ (range 30.0-47.5 kg/m^2^), HW=22.1 kg/m^2^ (range 18.5-24.0kg/m^2^) (P<0.001)  Right handed | | Block design | Standardised breakfast of 500kcal 6h prior | Salty and sweet high calorie foods  Salty and sweet low calorie foods |  |
| **Nummenmaa 2012^27^** | Case control | n=35.  OB n=19  HW n=15.  One HW excluded | No indication of gender  Age: OB=45.74±9.6 yrs, HW=45.75±10.44 yrs  BMI: OB=43.87±6.60 kg/m^2^, HW= 24.10±2.07 kg/m^2^ (P<0.001)  % body fat: OB=48.27±6.6%, HW= 29.37±6.37% (P<0.001) | | Block design | Fasting 3h | ‘Appetising’ foods  ‘Bland’ foods | Non-food control images |
| **Scharmuller 2012^26^** | Case control | N= 26  OB n=12  HW n=14 | Right handed, female  Age: HW=25.6±6.7yrs, OB=26.6± 4.5yrs  BMI: HW=20.6±1.3kg/m^2^, OB=31.5±5.2 kg/m^2^ (p<0.05)  3 of the OB group BMI in overweight category | | Event-related | Overnight fast | High calorie foods | 30 non-food images |
| **Ng 2011^45^** | Case control | n=34. Data from 4 I.e. final sample  OB n= 17,  HW n=17 | Female  Age: 20.1±1.4yrs (range 18-23yrs)  BMI: OB= 36.3±3.39 kg/m^2^, HW=22.1±1.04 kg/m^2^ | | Event-related | Fasting 4-6h (except water) | Coloured picture of chocolate milkshake | Glass of water or empty glass images |
| **Stoeckel 2008, 2009^28,^** ^46^ | Case control | n=24.  OB n=12  HW n=12 | Female  Age: OB=27.8±6.2 yrs, HW=28±4.4 yrs  BMI: OB=30.8-41.2 kg/m^2^, HW=19.7-24.5 kg/m^2^  Groups matched on phase of menstrual cycle | | Block design | Fasting 8h.  Abstain from alcohol for 24h prior; abstain from caffeine 3h prior. | High calorie foods, sweet and savoury  Low calorie foods | 84 non-food images |
| **Rothemund 2007**^47^ | Case control | N= 26  OB n=13  NW n=13 | Female  Age: HW=29±5.6yrs, OB=31±9.4yrs  BMI: HW=20.9±1.7 kg/m^2^, OB=36.3±4.8 kg/m^2^ (p<0.001)  Right handed. | | Block design | Fasting 1.5h | High calorie foods (≥4kcal/g)  Low calorie foods (1.5kcal/g) | 10 Food utensils.  10 non-food images |
| **Weight loss interventions** | | | | | | | | |
| **Frank 2014** ^48^ | Case control with pre- post- test outcomes | n=31  OB n=11  RYGB n=9  HW n=11 | Female  Age: HW 36.6±3.8yrs, RYGB 42.0±2.8yrs, OB 42.6±4.0yrs (P=0.412).  BMI: HW 21.4±0.5 kg/m^2^, RYGB 27.1±0.9 kg/m^2^, OB 49.2±0.8 kg/m^2^ (P<0.001).  Average time after RYGB 3.4±0.8yrs. | Block design | | Standardised liquid meal (246kcal) 30min prior to scan | High calorie food  Low calorie foods | Non-food pictures |
| **Goldman 2013** ^49^ | Case control | n=31  More successful weight loss (MS) n=24  Less successful weight loss (LS) n=7 | MS: female n=19, male n=12, LS: female n=7.  Age: MS: 46.58±11.36yrs, LS: 43.43±10.47yrs  Pre-surgery BMI: MS: 51.59±11.22 kg/m^2^, LS: 50.21±5.36 kg/m^2^.  Current BMI: MS 30.41±7.16 kg/m^2^, LS 38.16±3.69 kg/m^2^ (P<0.05).  % weight loss: MS: 40.81±8.21%, LS 23.60±6.46% (P<0.05).  >1 year post- surgery RYGB mean time since surgery 3.07yrs.  More successful= > 50% weight loss.  Matched for menstrual phase | Block design | | Fasting 4h | High calorie foods | Neutral non-food images |
| **McCaffery 2009^50^** | Case control | N=51  HW n=18  OB n=16  SWL n=17  1 HW and 1 OB excluded | HW female n=16, male n=2, OB female n=14, male n=2, SWL female n=15, male n=2  Age: HW=43.72±8.38yrs, OB=49.12±6.99yrs, SWL=48.47±11.37yrs  BMI: HW=21.70±1.98 kg/m^2^, OB=34.52±3.72 kg/m^2^, SWL=23.71±1.55 kg/m^2^ (P<0.001)  Groups defined by lifetime weight history. SWL loss group ≥13.6kg of weight loss and maintenance 3yrs. | Block design | | Fasting 4h (except water)  Limited alcoholic and caffeinated beverages previous 24h | High calorie foods  Low calorie foods | Non-food images |
| **Tregellas 2011^51^** | Case control | N= 32  HW n= 24  SWL n=18  1 HW and 1 SWL excluded | Participants in weight loss program with counselling from dietitians. Goal to maintain 8-10% weight loss.  HW female n=12, male n=12. SWL female n=10, male n=8  Age: HW=34.7±5.4yrs, SWL =35.2±5.7yrs  BMI: HW=21.6±1.7 kg/m^2^, SWL =27.5±2.6 kg/m^2^ (P<0.05)  Body fat %: HW=19.8±6.8, SWL =31.9±7.0 (P<0.05)  Weight loss 8.0±0.9%  Right handed, scanned in same phase of menstrual cycle | Block design | | 9 day diet phase: 6 day 'controlled' eucaloric diet phase and 3 day ad libitum diet phase (overfed by 30%)  Overnight fast on scan days | ‘Highly hedonic’ foods  ‘Neutral hedonic’ foods | Non-food images. |
| **Bruce 2011 ^52^** | Case series with pre-test post-test outcomes | n=12  2 loss to follow up i.e. n=10 | Female n=9, male n=3  Age: 40.10±10.27yrs  Pre-surgery BMI 40.61±1.96 kg/m^2^, post-surgery 36.14±2.32 kg/m^2^ (P<0.001). Weight loss after surgery 25.21±8.41%.  Gastric banging surgery candidates, right handed. | Block design | | Scan 1 (fasted): Fasting >4h  Scan 2 (satiated): following 500kcal meal. | Appetising food images | Animals, low level baseline blurred images |
| **Bruce 2014 ^53^** | Case series with pre-test post-test outcomes | n=31  Behavioural weight loss n=16  Bariatric weight loss n=15 | Behavioural: female n=11, male n=5, Bariatric: female n=12, male n=3.  Age: Behavioural 40.6±7.1yrs (range 23-52yrs), Bariatric 41.1±9.8yrs (range 21-56yrs)  BMI range 30-45 kg/m^2^  Weight loss % behavioural 10.8%, Bariatric 9.3% (p=0.32) | Block design | | Scan 1 (Fasted): 4h fast.  Scan 2 (satiated): standardised 500ckal meal. | Appetising foods | Animals, low level baseline blurred images |
| **Weygant 2013 ^54^** | Case series with pre-test post-test outcomes | n=16 | Female n=13, male n=3  Age: 43.0±12.2yrs (range 23.5-66.5yrs)  Pre-diet BMI: 34.5±3.2 kg/m^2^ (range 29.8-41.5 kg/m^2^).  BMI reduction following intervention 4.3±1.8 kg/m^2^  % weight loss 12.6% (range 2-23%)  Recruited from larger dietary study. | Event-related | | Nutritionist involved. Overnight fast >12h | High calorie foods  Low calorie foods | Eating related utensils  Non-food |
| **Murdaugh 2012**^55^ | Case series with pre-test post-test outcomes | N=38  OB n=21  Overweight n=4  HW n=13 | Participants in EatRight weight loss intervention  OB: female=19, male=6. HW: female=8, male=5  Age: OB=48.0±10.91yrs. HW=45.2±10yrs  Initial BMI: OB=32.86±3.82 kg/m^2^ (range 28.4-44.6 kg/m^2^), HW=22.64±1.58 kg/m^2^  Sig. decrease in weight in OB group following EatRight (-3.46% P<0.0001) and lost sig more weight than controls (0.80% P<0.004) | Block design | | Fasting 8h  Abstain from alcohol for 24h prior; abstain from caffeine 3h prior | High calorie foods, sweet and savoury  Low calorie foods | 84 non-food images |
| **Nock 2012^56^** | Case series with pre-test post-test outcomes | n=11.  Intervention n=8  Control n=3 | Females in endometrial cancer treatment group SUCCEED  Age: 54.4±7.4 yrs  BMI: 35.8±8.1kg/m^2^  Average weight loss after lifestyle intervention: -3.4%±2.7% | Block design | | Scan 1 (fasted): overnight fast  Scan 2 (satiated) 750kcal standardised meal prior | High calorie foods  Low calorie foods | Non-food images |
| **Ochner 2012** ^57^ | Case series with pre-test post-test outcomes | n=5 | Female  Age: 36±13yrs (range 21-54yrs)  Pre-operative BMI: 44.0±3.8 kg/m^2^ (range 39.1-48.1 kg/m^2^)  RYGB candidates, weight stable, right handed | Event-related | | Overnight 12 h fast.  Scan 1: 250kcal nutritionally complete beverage 45 mins prior to scan  Scan 2: 250mL water | High calorie foods >3.5kcal/g,  Low calorie foods <1kcal/g | N/A |
| **Ochner, 2012**^58^ | Case series with pre-test post-test outcomes | n=14.  N=2 excluded | Female gastric bypass candidates  Age: 36±10 yrs (range 20-54 yrs)  BMI: Pre-operative=45.4±4.4kg/m^2^ (range 40-54kg/m^2^). Post-operative= 39.8±3.7kg/m^2^ (P<0.0005) Surgical weight loss =11.8% original weight.  Scanned in same phase of menstrual cycle | Event-related | | Fasting 12h; 250kcal liquid meal 60 mins prior to scan | High calorie foods (>3.5kcal/g)  Low calorie foods (<1kcal/g) | Non-food images |
| **Ochner, 2011.** ^59^ | Case series with pre-test post-test outcomes | n=10  n=3 excluded | Female gastric bypass candidates  Age: 20-47 yrs (mean 35±9 yrs)  BMI: 40-54kg/m^2^ (mean 45±5kg/m^2^)  Right handed | Event-related | | Fasting 12h; 250kcal liquid meal 60 mins prior to scan | High calorie foods (>3.5kcal/g)  Low calorie foods (<1kcal/g) | Non-food images |
| **Healthy weight** | | | | | | | | |
| **Benedict 2012 ^60^** | Randomised crossover design | n=12 | Healthy males  Age 23.3±0.6yrs  BMI: 22.5±0.5 kg/m^2^ | Block design | | Standardised dinner (700kcal). 350ml standardised breakfast (125kcal curdled milk) | High calorie food  Low calorie food | N/A |
| **Karra 2014 ^61^** | Counterbalanced crossover | N=24  TT genotype n=12  AA genotype n=12 | Males  Age: TT genotype 22.1±1.0, AA genotype 23.0±0.8  BMI: TT genotype 21.6±0.3, AA genotype 22.3±0.15  Right handed | Block design | | Scan 1 (fasted): overnight fast  Scan 2 (satiated): Standard meal 1840kcal | High calorie food  Low calorie food | Non-food |
| **Kroemer 2012 2013 ^62, 63^** | Double blind randomised crossover design | n=30, 4 excluded i.e. n=26 | Female n=13, male n=13.  Mean age 24.4±3.4yrs.  BMI 21.1±2.0 kg/m^2^  Menstrual cycle controlled | Block design | | Scan 1: Overnight fast  Scan 2: 300ml drink containing 75g glucose | Highly palatable foods | Scrambled images |
| **Evero, 2012^64^** | Within subjects crossover design | n=30 | Male n=17, female n=13.  Age: 22.2±0.7 yrs  BMI: 23.6±0.4 kg/m^2^  Body fat % 16.7±1.3%  Right handed, scanned in same phase of menstrual cycle. | Block design | | Fasting 8-12h  Refrain from alcohol and caffeine for 24h prior | Low energy foods  High energy foods | Non-food images |
| **Born, 2011^65^** | Within participants crossover design | n=15 | Right handed healthy females  Age: 21.5±0.4 yrs  BMI: 22.2±0.2 kg/m^2^ (range 19.5-25.5kg/m^2^) | Event-related | | Scan 1 (fasted): Overnight fast (>10h); Restrain from alcohol and caffeine  Scan 2 (satiated): breakfast provided prior to scan; Second meal given after second scan | High and low calorie foods |  |
| **Born 2010^66^** | Within participants crossover design | n=10.  1 participant data excluded | Right handed females  Age: 24.1±1.1 yrs  BMI: 21.5±0.7kg/m^2^ | Event-related | | Scan 1: Fasted state  Scan 2 (satiated): breakfast provided before second scan | High and low calorie foods |  |
| **Cornier 2010**^67^ | Randomised crossover design | n=43 | Females n=22, males n=21.  Age 25-45 yrs  Age: Female 35.8±5.4yrs, males: 34.2±5.7yrs  BMI: female 24.3±4.1 kg/m^2^, male 24.2±3.0 kg/m^2^  Body fat%: female 33.8±4.6%, male 18.5±5.0%  Healthy, right handed | Block design | | 9 day diet phase: 6 day 'controlled' eucaloric diet phase and 3 day ad libitum diet phase (overfed by 15%)  Overnight fast on scan days No alcohol or calorie containing beverages. | ‘Highly hedonic’ foods  ‘Neutral hedonic’ foods | Neutral non-food images |
| **Frank 2010**^68^ | Within subjects crossover design | n=12 | Male n=6, female n=6  Age: 27.42y±4.14yrs  BMI: 22.4±2.55 kg/m^2^  Healthy, right handed | Block design | | Scan 1 (fasted): >3h fast  Scan 2 (satiated): lunch prior to scan | 36 high calorie foods  36 low calorie foods | 72 non-food related images |
| **Goldstone 2009^69^** | Randomised crossover design | n=20 | Male n=10, female n=10  Age: 26±1 (range 19-35)  BMI: 22.1±0.5 kg/m^2^ (range 18.2±27.1 kg/m^2^). One subject with BMI>24.9  Right handed, healthy, stable body weight | Block design | | Scan 1 (fasted): overnight fast (except water)  Scan 2 (satiated): consumption of breakfast prior to scan  Avoid alcohol previous day. | High calorie foods  Low calorie foods | Non-food images  Blurred images. |
| **Coletta 2009^70^** | Within participants crossover design | n=19  Restrained eaters n=9  Unrestrained n=10 | Females  Age: Restrained=19.7±1.09yrs, Unrestrained=20.6±2.71yrs  BMI: Restrained=22.1±0.59 kg/m^2^, Unrestrained=21.5±1.86kg/m^2^  Healthy, right handed, predominantly Caucasian. | Event-related | | Scan 1 (fasted): fasting 8h  Scan 2 (satiated): 2 cans of vanilla Ensure (500kcal total) prior to scan | ‘Highly palatable’ foods  ‘Moderate palatable’ foods | 78 non-food images |
| **Siep 2009^71^** | Within subjects crossover design | n=12 | Healthy, right handed females  Age: 19.3±0.9 yrs.  BMI: 21.5±1.9kg/m^2^(range 18.5-25kg/m^2^)  Not currently dieting or restrained eaters | Block design | | Fasting condition: fasting (except water) 18h  Satiated condition: 500kcal standardised 30min prior to scan | High calorie foods  Low calorie foods | 15 Non-food images |
| **Fuhrer 2008^72^** | Within participants crossover design | n=12 | Healthy males  Age: 26.42 yrs (range 21-29 yrs)  BMI: 18.4-24.7kg/m^2^ | Block design | | Scan 1 (fasted): fasting 14h (non-caloric beverages only)  Scan 2 (satiated): meal1h prior to scan | Food images (no indication on calorific content) | 50 non-food images. |
| **Cornier 2007^73^** | Randomised crossover design | n=25 | Female n=13, male n=12.  Age: Female 35.6±6.2 yrs, male 33.8±4.7yrs  BMI: female 21.0±1.3 kg/m^2^, male 22.0±1.9 kg/m^2^  Body fat %: female 28.8±3.4%, male 16.4±4.0%  Right handed, healthy | Block design | | 9 day diet phase: 6 day 'controlled' eucaloric diet phase and 3 day ad libitum diet phase (overfed)  Overnight fast on scan days No alcohol or calorie containing beverages. | ‘Highly hedonic’ foods  ‘Neutral hedonic’ foods | Neutral non-food images |
| **Uher 2006** ^74^ | Within participants crossover design | n=18 | Female n=10, male n=8  Age: females 28.4±8.4 yrs (range 20-44), males 29.4±7.8 (range 23-42)  BMI: females 22.5±2.8 kg/m^2^(range 19.9-27.4), males 22.4±2.5 kg/m^2^(range 17.0-24.7) | Event-related | | Scan 1 (Fasted): Fasting 24h (excluding water)  Scan 2 (satiated): meal 3h prior to scan  Abstain from alcohol for 24h and caffeinated beverages on scanning day | Food images (sweet and savoury); no indication of calorific content | 20 non-edible objects images |
| **Demos 2011**^75^ | Case control | n=100  Dieters n=50  Non dieters n=50  9 participants data excluded | Female.  Age: 19.50 yrs (range 18-35 yrs)  BMI: Dieters=23.5±2.7kg/m^2^, Non-dieters=22.6±2.4kg/m^2^  Mean restraint dieters=19.6±2.8, non-dieters=8.7±2.8 (P<0.0001) | Event-related | | Fasting 2h; no alcohol, caffeine or smoking for 2h prior | Appetising food | 300 non-food images |
| **Rolls 2007^76^** | Case control | n=16  Cravers n=8  Non cravers n=8 | Healthy females.  Age: range 20-30 yrs.  BMI: Cravers=22.1 kg/m^2^, Non-cravers=23.0 kg/m^2^ (ns) | Event-related | | No choc for 2 days prior to scan; small lunch prior to scan | Dark chocolate image |  |
| **Grabenhorst 2013 ^77^** | Cross sectional | n=13 | Female n=7, males n=6.  Age 24.2yrs (range 22-27yrs)  BMI range 22-25 kg/m^2^  Right handed | Event-related | | Fasting 3h | Common food stimuli labelled as ‘tasty’ or ‘healthy’ |  |
| **Hollman 2012^78^** | Cross sectional study | n=20. 3 excluded I.e. n=17 | Healthy, right handed females  HW n=17, overweight n=3  Age: 25.3±3.2yrs (range 20-30 yrs)  BMI: 25.1±3.5 kg/m^2^(range 20.2±31.2 kg/m^2^)  Scanned in same menstrual phase | Event-related | | Fasting ≥6h | High calorie ‘tasty’ foods  High calorie ‘non tasty’ foods. All >190kcal/100g. |  |
| **Lawrence 2012^79^** | Cross sectional study | n=25 | Healthy, right handed females.  Age: 21.4±3.2 yrs (range 18-29yrs)  BMI: 21.7±0.6 kg/m^2^ (range 17.1± 30.1 kg/m^2^) (2 underweight and 3 overweight). | Event-related | | Fasting 6h | Food images: high and low calorie, salty and sweet, bland and highly appetising. | 45 non-food images |
| **Siep 2012**^80^ | Cross sectional study | n=14 | Healthy, right handed females  Age: 21.5±1.5 yrs  BMI: 21.5±1.9 kg/m^2^(range 18.5-25 kg/m^2^)  Not currently dieting or restrained eaters | Event-related | | Fasting 4h (except water) | High calorie foods, sweet and savoury |  |
| **Gearhardt 2011^29^** | Cross sectional study | n=48 | Female  Age: 20.8±1.31yrs  BMI: 28.0±3.0 kg/m^2^ (range 23.8±39.2 kg/m^2^)  High food addiction ≥3 YFAS symptoms, ≤1 symptom low FA | Event-related | | Fasting 4-6h prior | Milkshake | Glass of water images |
| **Schur 2009^81^** | Cross sectional study | n=10 | Female  Age: 29.4±12.1yrs (range 20.4-49.5yrs )  BMI: 23.2±2.0 kg/m^2^ (range 20.4-26.8 kg/m^2^)  No history of weight loss surgery or current dieting. | Block design | | Instructed to eat normal meal 2-4h prior to scan | ‘Fattening’ foods  ‘Non-fattening’ foods | Non-food images |
| **Passamonti 2009^82^** | Cross sectional study | n=21 | Female n=10, males n=11  Age: 25.3yrs (range 19-39)  BMI: 24±4.6 kg/m^2^  Healthy, right handed | Block design | | Fasting ≥2h | Highly appetising foods  Bland foods. |  |
| **Killgore 2006 ^83-85^** | Cross sectional study | n=13 | Right handed, female  Age 21-28 yrs (range 23.5±2.1yrs)  BMI: 22.1±2.4m^2^ | Block design | | Fasting 90min | Low calorie foods  High calorie foods | Food utensils  Non-food images |
| **Porubska 2006^86^** | Cross sectional study | n=12 | Male n=7, female n=5.  BMI: 21.8±1.96 kg/m^2^  Age: 27.17±5.36yrs | Event-related | | Fasting ≥5h | High and low calorie food stimuli | 64 food neutral images |
| **Simmons 2005^87^** | Cross sectional study | n=12 | Male n=6, female n=6.  Age: 29.8±1.8yrs  BMI: 21.8±0.8kg/m^2^  Healthy, right handed. | Block design | | Fasting 12h  1 subject non-compliant but included in analyses | High calorie foods  Low calorie foods | Non-food images |
| **Overweight/obese** | | | | | | | | |
| **Geliebter 2013 ^88^** | Within participants crossover design | n=31, | Female n=14, male n=17.  Age: male 35±0.9yrs, female 35±6.9yrs  BMI: male 36.2±5.5 kg/m^2^, female 36.9±5.6 kg/m^2^. | Block design | | Meal prior to fast (1000kcal). 12h overnight fast.  Scan 1 (Fasted): 750mL water  Scan 2 (satiated): 750mL shake | High energy dense food >3.5kcal/g  Low energy density foods <1kcal/g | Non-food |
| **Lundgren 2013 ^89^** | Within participants crossover design | n=14  Evening hyperphagia (EH) n=7  Controls n=7 | EH: female n=6, BMI 37.9 kg/m^2^, age 33.6yrs  Controls matched to EH  EH definition: consume >25% energy after dinner | Block design | | Scan 1 (fasting): 4 h fast  Scan 2 (satiated): following 500cal meal | Food | Animals, blurred baseline images |
| **Tryon 2013** ^90^ | Within participants crossover design | n=30 | Female  Age: 39.7±2.3yrs.  BMI: 25.6±0.9 kg/m^2^ (range 18-39 kg/m^2^).  Right handed, menstrual phase controlled | Event-related | | Standardised lunch, fasting 2.5h.  Buffet following scan, dietitian involved | High calorie foods  Low calorie foods | Non-food items |
| **Van Vugt 2012 ^91^** | Case control | n=17, 1 excluded i.e. n=16  Insulin sensitive (IS) n=9  Insulin resistant (IR) n=7. | Right handed females with polycystic ovarian syndrome  Age: total: 27.8±5.72yrs (18-39yrs), IS: 26.4±5.27yrs (20-36yrs), IR: 29.6±6.19yrs (18-39yrs).  BMI: total: 36.6±9.61 kg/m^2^ (23.5-55.5 kg/m^2^), IS: 32.4±7.94 kg/m^2^ (23.5-47.6 kg/m^2^), IR: 42.1±9.23 kg/m^2^ (26.7-55.5 kg/m^2^) (P=0.02) | Block design | | Fasting >5h | High calorie sweet and savoury foods  Low calorie water and fibre based foods | Non-food items |
| **Luo 2012 ^92^** | Cross sectional | n=13 | Female,  BMI: 34±4 kg/m^2^ (range 28-40 kg/m^2^)  Age 23±2yrs  Right handed, menstrual phase controlled. | Block design | | 10-12h fast overnight | High calorie food  Low calorie food | Non-foods |

^a^ Visual cue classification has used the authors description where possible, ^b^ only HW and OB results reported

OB= Obese, OW=overweight HW= healthy weight, SWL= OB participants who had successfully lost weight, BMI= body mass index, RYGB= Roux-en-Y gastric bypass
