# Supplementary material for: Neural Responses to Visual Food Cues According to Weight Status: A Systematic Review of Functional Magnetic Resonance Imaging Studies
Source: Front Nutr. 2014 Jul 9;1:7. doi: 10.3389/fnut.2014.00007 (PMC4428493; doi:10.3389/fnut.2014.00007)
Supplement: Supplementary file 2 [file Data_Sheet_2.DOCX]

| ***Reference***  Table 2: Critical Appraisal | *Was the research question clearly stated?*  **Table 2:** Study Quality of included studies investigating neural responses to visual food cues | *Was the selection of study subjects free from bias?** | *Were study groups comparable?** | *Were withdrawals described?* | *Was blinding used to prevent bias?* | *Were interventions described?** | *Were outcomes clearly defined?** | *Was the statistical analysis appropriate?* | *Were conclusions supported by results?* | *Is bias due to study’s funding/ sponsorship?* | *Overall Quality* |
| --- | --- | --- | --- | --- | --- | --- | --- | --- | --- | --- | --- |
| **Frank 2014 ^48^** | Y | Y | Y | UC | UC | Y | Y | Y | Y | Y | P |
| **Bruce 2014 ^53^** | Y | Y | Y | UC | UC | Y | Y | Y | Y | Y | P |
| **Connolly 2013 ^33^** | Y | Y | Y | Y | Y | Y | Y | Y | Y | Y | P |
| **Cornier 2013 ^34^** | Y | Y | UC | UC | UC | Y | Y | Y | Y | Y | 0 |
| **Garcia-Garcia 2013 ^40^** | Y | Y | Y | Y | UC | Y | Y | Y | Y | Y | P |
| **Geliebter 2013 ^88^** | Y | Y | UC | UC | UC | Y | Y | Y | Y | Y | 0 |
| **Goldman 2013 ^49^** | Y | Y | Y | UC | UC | Y | Y | Y | Y | Y | P |
| **Grabenhorst 2013 ^77^** | Y | Y | Y | UC | UC | Y | Y | Y | UC | UC | P |
| **Jastreboff 2013 ^41^** | Y | Y | Y | UC | UC | Y | Y | Y | Y | Y | P |
| **Karra 2013 ^61^** | Y | Y | Y | Y | UC | Y | Y | Y | UC | Y | P |
| **Kroemer 2013 ^63^** | Y | Y | NA | Y | UC | Y | Y | Y | Y | Y | P |
| **Kullmann 2013 ^42^** | Y | UC | Y | Y | UC | Y | Y | Y | Y | Y | 0 |
| **Lee 2013 ^36^** | Y | Y | Y | Y | UC | Y | Y | Y | Y | Y | P |
| **Lundgren 2013 ^89^** | Y | Y | Y | UC | UC | Y | Y | Y | UC | Y | P |
| **Luo 2013 ^92^** | Y | UC | NA | UC | UC | Y | Y | Y | Y | Y | 0 |
| **Martens 2013 ^37^** | Y | Y | Y | Y | UC | Y | Y | Y | Y | Y | P |
| **Tryon 2013 ^90^** | Y | Y | Y | UC | UC | Y | Y | Y | Y | Y | P |
| **Van Vugt 2013 ^91^** | Y | Y | Y | Y | UC | Y | Y | Y | Y | Y | P |
| **Weygandt 2013 ^54^** | Y | Y | NA | UC | UC | Y | Y | Y | Y | Y | P |
| **Benedict 2012 ^60^** | Y | Y | Y | UC | UC | Y | Y | Y | Y | Y | P |
| **Dimitropoulos 2012^38^** | Y | Y | Y | UC | UC | Y | Y | Y | Y | Y | P |
| **Evero, 2012^64^** | Y | Y | NA | UC | UC | Y | Y | Y | Y | UC | P |
| **Frankort 2012^43^** | Y | Y | Y | Y | UC | Y | Y | Y | Y | Y | P |
| **Grosshans 2012 ^44^** | Y | Y | Y | Y | UC | Y | Y | Y | Y | UC | P |
| **Ho 2012 ^35^** | Y | Y | Y | Y | UC | Y | Y | Y | Y | Y | P |
| **Hollman 2012^78^** | Y | Y | NA | UC | UC | Y | Y | Y | Y | Y | P |
| **Holsen 2012^39^** | Y | Y | Y | Y | UC | Y | Y | Y | Y | Y | P |
| **Kroemer 2012 ^62^** | Y | Y | NA | UC | UC | Y | Y | Y | Y | Y | P |
| **Lawrence 2012^79^** | Y | Y | Y | UC | UC | Y | Y | Y | Y | Y | P |
| **Murdaugh 2012**^55^ | Y | Y | Y | UC | UC | Y | Y | Y | Y | Y | P |
| **Nock 2012^56^** | Y | Y | UC | N | UC | Y | Y | Y | Y | Y | 0 |
| **Nummenmaa 2012^27^** | Y | Y | Y | Y | UC | Y | Y | Y | Y | Y | P |
| **Ochner 2012 ^57^** | Y | Y | NA | UC | UC | Y | Y | Y | Y | Y | P |
| **Ochner, 2012**^58^ | Y | Y | NA | Y | UC | Y | Y | Y | Y | Y | P |
| **Scharmuller 2012^26^** | Y | Y | Y | UC | UC | Y | Y | Y | Y | UC | P |
| **Siep 2012**^80^ | Y | Y | NA | UC | UC | Y | Y | Y | Y | UC | P |
| **Born, 2011^65^** | N | Y | NA | UC | UC | Y | Y | Y | UC | Y | P |
| **Bruce 2011^52^** | Y | Y | NA | UC | UC | Y | Y | Y | Y | Y | P |
| **Demos 2011**^75^ | Y | Y | Y | Y | UC | Y | Y | Y | Y | Y | P |
| **Gearhardt 2011^29^** | Y | Y | Y | Y | UC | Y | Y | Y | Y | Y | P |
| **Ochner, 2011.** ^59^ | Y | Y | NA | Y | UC | Y | Y | Y | Y | Y | P |
| **Ng 2011^45^** | Y | Y | Y | Y | UC | Y | Y | Y | Y | Y | P |
| **Tregellas 2011^51^** | Y | Y | NA | Y | UC | Y | Y | Y | UC | Y | P |
| **Born 2010^66^** | Y | Y | NA | Y | UC | Y | Y | Y | UC | Y | P |
| **Cornier 2010**^67^ | Y | Y | NA | Y | UC | Y | Y | Y | Y | Y | P |
| **Frank 2010**^68^ | Y | Y | NA | UC | UC | Y | Y | Y | Y | Y | P |
| **Coletta 2009^70^** | Y | Y | NA | UC | UC | Y | Y | Y | Y | UC | P |
| **Goldstone 2009^69^** | Y | Y | NA | Y | UC | Y | Y | Y | Y | Y | P |
| **Schur 2009^81^** | Y | Y | NA | Y | UC | Y | Y | Y | Y | Y | P |
| **Siep 2009^71^** | Y | Y | NA | UC | UC | Y | Y | Y | UC | UC | P |
| **McCaffery 2009^50^** | Y | Y | Y | Y | UC | Y | Y | Y | Y | Y | P |
| **Passamonti 2009^82^** | Y | Y | NA | Y | UC | Y | Y | Y | Y | Y | P |
| **Stoeckel 2009^46^** | Y | Y | Y | Y | UC | Y | Y | Y | Y | Y | P |
| **Fuhrer 2008^72^** | Y | Y | NA | UC | UC | Y | Y | Y | Y | Y | P |
| **Stoeckel 2008^28^** | Y | Y | Y | Y | UC | Y | Y | Y | Y | Y | P |
| **Cornier 2007^73^** | Y | Y | NA | Y | UC | Y | Y | Y | Y | Y | P |
| **Rolls 2007^76^** | Y | Y | N | UC | UC | Y | Y | Y | UC | UC | P |
| **Rothemund 2007**^47^ | Y | Y | Y | UC | UC | Y | Y | Y | Y | Y | P |
| **Killgore 2006^85^** | Y | Y | NA | UC | UC | Y | Y | Y | Y | UC | P |
| **Porubska 2006^86^** | Y | Y | NA | UC | UC | Y | Y | Y | UC | Y | P |
| **Uher 2006 ^74^** | Y | Y | NA | Y | UC | Y | Y | Y | Y | Y | P |
| **Killgore 2005^84^** | Y | Y | NA | UC | UC | Y | Y | Y | UC | Y | P |
| **Simmons 2005^87^** | Y | Y | NA | UC | UC | Y | Y | Y | Y | Y | P |
| **Killgore 2003^83^** | Y | Y | NA | Y | UC | Y | Y | Y | Y | UC | P |

*= Important criteria, N= No, N/A= Non applicable, 0=Neutral, quality P=Positive quality, UC= Unclear, Y=Yes
