# Supplementary material for: Neural Responses to Visual Food Cues According to Weight Status: A Systematic Review of Functional Magnetic Resonance Imaging Studies
Source: Front Nutr. 2014 Jul 9;1:7. doi: 10.3389/fnut.2014.00007 (PMC4428493; doi:10.3389/fnut.2014.00007)
Supplement: Supplementary file 3 [file Data_Sheet_3.DOCX]

**Table 3:** Outcome measures and results of included studies investigating neural responses to visual food cues by weight status

| **Author** | **Outcome measures** | **fMRI acquisition** | **fMRI Preprocessing and analysis** | **fMRI outcomes^a^** | **Conclusions** | **Limitations** |
| --- | --- | --- | --- | --- | --- | --- |
| **Healthy weight and overweight/obese** | | | | | | |
| **Connolly 2013 ^33^** | Anxiety and Depression scale; Speilberger State And Trait Anxiety Inventory; Appetite and image ratings | 1.5T | SPM5, ROI P<0.05 FWE corrected | Food>control images all subjects ↑ activity in amygdala, hippocampus, thalamus and and insula. OB>HW high cal>non-nutritive beverage ↑ in activity ant insula, ACC, amygdala, hippocampus, visual cortex. Greater hunger score correlated with ↑activity in left post insula. OB>lean ↑ activation of right ant insula correlated with hunger. | Greater responses in affective and memory related brain regions in OB when viewing food images after drinking sucrose drink | Female only, small sample size, meals not tailored to requirements |
| **Cornier 2013 ^34^** | Anthropometrics; Hunger ratings | 3.0T, parallel to planum sphenoidale | SPM8, , whole brain voxel wise threshold <0.01 cluster level FDR q<0.05 | Obese resistant (OR) fasted high hedonic food> non-food ↑ activation in insula, inf PFC, somatosensory cortex, visual cortex. OR satiated vs fasted ↓ activation to food cues in the insula, inf PFC, somatosensory cortex, visual cortex, putamen, post cingulate cortex. Obese prone (OP) fasted high hedonic>non-food ↑ activation of insula OFC, somatosensory cortex, post cingulate cortex, parietal cortex, visual cortex. OP satiated> fed ↑ activation to food in the middle PFC, ant PFC, let PFC, insula/inf PFC. OR>OP fed state ↑ activation in insula, inf PFC, medial PFC | Neuronal response to food cues in the fasted state is attenuated in OR but not OP | Self-described as OP or OR, differences in baseline BMI |
| **Ho 2012** ^35^ | Food-related problem questionnaire; Anthropometrics, Food preference; Hunger ratings | 4.0T, transverse plane parallel to AC-PC line | BrainVoyagerQX, ROI P<0.005 min cluster >5 contiguous voxels. Whole brain P<0.001 cluster threshold >5 FDR P<0.05. | Pre-meal HW high cal vs non-food activation of the amygdala positively correlated with satiety (r=0.70, p=0.005). Pre-meal OW/OB low cal vs non-food activation of the dlPFC negatively correlated with satiety (r=-0.56, p=0.007). Post- meal, OW/OB low cal vs non-food activation of the putamen and amygdala positively correlated with satiety (r=0.64 p=0.002 and r=0.49 p=0.02). | Neural activation to food cues correlated with satiety depending on weight status, foods shown, and satiety status. | Small sample |
| **Lee 2013 ^36^** | Anthropometrics; TFEQ; 24 h recall; Appetite and mood ratings; Image ratings | 3.0T, oblique transverse plane parallel to AC-PC line | BrainVoyagerQX,  whole brain voxel threshold P<0.001. Cluster size threshold FWE P<0.05 | Low disinhibition (LD) satiated vs fasted ↓activation in the post vmPFC. High disinhibition (HD) satiated>fasted ↑ activation in the post vmPFC. LD Satiated vs fasted ↓activation in ant vmPFC. HD satiated> fasted ↑activation in ant vmPFC. | LD have devaluation of food when satiated leading to overeating. | Male only |
| **Martens 2013 ^37^** | Appetite and image ratings | 3.0T | BrainVoyagerQX 2.3., Whole brain voxel threshold p=0.01. Cluster level false positive rate of 5%. | Fasted food>non-food ↑ activation sup/middle/medial frontal gyrus (PFC), insula, cingulate gyrus, thalamus. Fasted food>non-food positively associated with BMI in the ACC (P<0.004, r=0.47). Satiated food>non-food ↑ activation insula. Satiated food>non-food negatively correlated with BMI in the sup frontal gyrus (PFC) (r=-0.45 P<0.005) | OW had more pronounced food reward anticipation related brain signalling in the fasted state than HW | Small sample, female only, |
| **Dimitropoulos 2012^38^** | Caloric intake;  Hunger ratings;  Food preferences | 4.0T; transverse plane, parallel to AC-PC line. | BrainVoyagerQX  P<0.01 uncorrected  FWE p<0.05  Minimum cluster correction 7-12 voxels | Pre-meal OB > HW ↑ activation foods>control images in ant PFC, sup frontal gyrus and cerebellum. Pre-meal HW >OB ↑ activity high > low-cal foods in L postcentral gyrus, insula, parahippocampal gyrus and cerebellum. Post- meal OB>HW ↑ activation food>control images in frontal areas and corticolimbic reward systems including OFC, caudate, ant cingulate. | High-cal foods continue to activate reward regions in OB following meal. | States not counterbalanced, handedness not controlled, stage of menstrual cycle not controlled, small sample. |
| **Holsen 2012^39^** | TFEQ | 3.0T; coronal plane | SPM8 ROI  Uncorrected p<0.05, FWE P<0.02, 2-4 contiguous voxels | OB> control participants exhibited ↑ activations to food vs non-food stimuli in the hypothalamus, amygdala, PFC, and OFC when fasted and the hypothalamus and dlPFC in the satiated state. HW> OB ↑ activation in the OFC to high cal foods in satiated state ^b^ | BMI affects brain responsivity to food cues. | Meal size not tailored to individual’s needs; hunger not measured, small sample size, no gender analysis |
| **Garcia Garcia 2013** ^40^ | Hunger ratings | 3.0T, transverse plane, whole brain | FSL software, corrected cluster threshold P<0.05 | Both obese & normal weight displayed activation of visual and frontal areas and deactivation of default mode network (DMN) in response to rewarding stimuli (both food and non-food).The strength of connectivity within these networks was weaker in obese individuals. Both obese and normal weight displayed activation of the DMN & deactivation of occipital and frontal areas to neutral stimuli. The strength of connectivity was reduced in obese individuals. | OB showed lower connectivity in areas involved in the integration of reward. | Cannot elucidate the contributions of individual parts of networks studied |
| **Jastreboff 2013** ^41^ | Fasting plasma insulin and glucose; Hunger ratings; Anxiety ratings | 3.0T, transverse plane parallel to AC-PC line | SPM5 Cluster threshold P<0.01 k=54 voxels. Whole brain FWE | OB food>neutral ↑ activation putamen, insula, thalamus, hypothalamus, parahippocampus, inf frontal gyrus, middle temporal gyrus. OB stress>relaxing ↑ activation insula, putamen, inf frontal gyrus, and middle temporal gyrus. Insulin resistance positively correlated with corticolimbic-striatal activation. | Insulin resistance in OB may contribute to dysregulated eating and predispose individuals to overeat | Blood tests taken at different time to scans, imprecise measure of insulin resistance |
| **Kullmann 2013 ^42^** | Hunger ratings; BGL; Plasma insulin; Profile of Mood States; Patient health questionnaire; TFEQ | 3.0T | SPM5, ROI P<0.05 FWE corrected | Inf occipital gyrus had ↓ functional connectivity in OW/OB>HW. ACC sup medial frontal gyrus increased functional connectivity in OW/OB > HW. Precuneus ↑ connectivity in OW/OB >HW. Connectivity between temporal visual association and salience networks ↑ in OW/OB >HW. | Changes in brain connectivity for OW/OB may lead to overconsumption | Small sample size, menstrual cycle not controlled |
| **Frankort 2012^43^** | Hunger;  Restraint scale;  PANAS;  Palatability ranking | 3.0T; oblique transverse plane (negative 30^0^ tilt) | BrainVoyager QX  Whole brain and ROI P<0.01 uncorrected, 64mm^3^ minimum cluster size threshold | Reward related regions activated to high cal foods including the amygdala, post fusiform gyrus, dlPFC, ant insula, OFC, VTA, substantia nigra, parahippoocampal gyrus, medial PFC, ant midcingulate gyrus, paracingulate gyrus. Imagining taste of food OB > HW ↑ reward related activation. Unbiased viewing HW>OB had ↑ activation to high-cal foods in reward regions. | Reward related activation dependent on food stimuli, cognitive strategies and BMI. | Conditions not counterbalanced, compliance not assessed , OB underrepresented, females only |
| **Grosshans 2012**^44^ | Leptin plasma concentration;  TFEQ;  Craving ratings | 3.0T; oblique transverse plane (30^0^ clockwise tilt from AC-PC line) | SPM5 Whole brain  FWE P<0.05  Min cluster size= 10 contiguous voxels | ↑ activation to food cues > neutral cues in the occipital lobe, inf frontal gyrus, sup parietal lobule, cingulate gyrus in OB vs HW. Positive correlation between activation in the VS and BMI (r=0.47, p=0.001) and leptin concentration (r=0.27, P=0.04). | Leptin plays a role in modulating the responsivity of the reward pathways to food cues. | Small ROI analysis, small sample size |
| **Nummenmaa 2012^27^** | Hunger ratings;  Valence of stimuli;  Positron emission tomography (PET);  PPI brain connectivity | 1.5T | SPM 5 Whole brain, ROI for PET and PPI  FDR p<0.05, P<0.005 uncorrected, min cluster 20 contiguous voxels | OB > HW ↑ responses to all foods in the amygdala, hippocampus, PCC, supramarginal gyrus, and fusiform gyrus. OB> HW↑ response to appetising> bland foods in the caudate. HW > OB ↑ response to appetising vs bland foods in the insula, OFC, lateral frontal cortex, sup parietal lobule, lateral frontal cortex and sup temporal gyrus. Sig. stronger connectivity OB>HW between the caudate nucleus and the basolateral amygdala and the post insula. | Obesity is associated with modified responses of the reward system and enhanced sensitivity to external food cues | No indication of participant gender. |
| **Scharmuller 2012^26^** | Hunger ratings;  Appetite ratings | 3.0T; oblique transverse plane (negative 10^0^ tilt from AC-PC line) | SPM8 ROI  P<0.005 uncorrected, min 5 contiguous voxels. P<0.05 FWE.  Specific hypotheses P<0.001 uncorrected | OB passive viewing of food > control images↑ activation in insula and medial and lateral OFC. OB downregulation of appetite > passive viewing of food images ↑ activation in dlPFC. HW upregulate appetite > passive viewing of food images ↑activation in insula and dorsal striatum. HW downregulating appetite > upregulating appetite viewing food images ↑ dlPFC, dmPFC, OFC, insula, dorsal striatum. | OB people may make a more strenuous but unsuccessful effort to reduce food craving. | Small sample size, female only, OB category also contained overweight BMI. |
| **Ng 2011^45^** | Fullness ratings; Ratings of food desire;  Liking ratings | 3.0T; transverse plane, parallel to AC-PC line | SPM5 Whole brain and ROI  P<0.005 corrected  Threshold >3 contiguous voxels. FDR P<0.05. | OB> HW ↑ activation in post cingulate gyrus and caudate to high fat milkshake image vs tasteless solution. OB >HW ↑activation in Rolandic operculum, hippocampus and parahippocampal gyrus to low fat milkshake image vs tasteless solution. OB >HW ↑ activation in Rolandic operculum, inf frontal gyrus and frontal operculum to high vs low fat milkshake image. | OB may overeat low fat foods to compensate for abnormal reward responses. | Only one food stimulus, hunger ratings only in subset of participants, female only, small sample. |
| **Stoeckel 2008, 2009 ^28, 46^** | Hunger ratings;  Valence ratings of stimuli;  PANAS;  Path analysis | 3.0T; transverse plane | SPM2 ROI  ROI P<0.01, corrected, min 7 contiguous voxels | OB> HW High cal>non-food ↑ activation in the medial and lateral OFC, medial PFC, ACC, insula, NAcc, amygdala, ventral palladium, hippocampus, caudate and putamen. HW> OB High cal> non-food ↑ activation in medial PFC. OB>HW low cal> non-food ↑ activation in caudate and putamen. HW>OB low cal> non-food ↑ activation in lat OFC, med PFC and ACC. Path analysis OB vs controls ↓ activation from amygdala to OFC and NAcc; ↑ connectivity from the OFC to NAcc compared to controls | ↑ activation to high-cal foods in OB in brain regions associated with motivational and emotional responses | Signal dropout in the med OFC, small sample size, VTA not included due to methodological limitations, female only |
| **Rothemund 2007**^47^ | Appetite ratings | 1.5T; transverse plane, parallel to AC-PC line. | SPM2 whole brain  P<0.001 uncorrected, min cluster size 5 contiguous voxels. P<0.05 corrected | OB> HW ↑ activation of putamen, caudate body, ant insula, hippocampus and parietal lobule to high-cal foods. OB> HW ↑ activations in sup frontal, middle and inf frontal gyrus, middle occipital gyrus and sup temporal gyrus to low cal foods. BMI sig. correlated with signal changes in the putamen (right: r=0.56; left: r=0.52), caudate body (r=0.53), ant insula (r=0.55), claustrum (r=0.52), post cingulate cortex (r=0.56), lateral globus pallidus (r=0.52), postcentral gyrus (r=0.54), lateral OFC (r=0.53). | Food images activate reward systems which may contribute to overeating and obesity. | Handedness not controlled, no description of food stimuli used, female only, small sample |
| **Weight loss intervention studies** | | | | | | |
| **Frank 2014^48^** | Hunger ratings; Prospective food consumption; TFEQ;  Body composition | 1.5T | SPM8, ROI P<0.05 FEW corrected | OB ↑ activation in the cerebellum and ↓activation in the fusiform compared to HW and RYGB. All participants high cal> low cal ↑ activation in OFC, insular cortex, amygdala, ACC and fusiform gyrus. All participants low cal> non-food ↑ activation in fusiform gyrus. OB low cal>high cal ↑ activation while high cal>low cal ↑ activation in hypothalamus in HW and RYGB. | RYGB activity differed from OB but similar to HW i.e. alterations in OB brain activity may recover following RYGB. | Female only, small sample, |
| **Goldman 2013** ^49^ |  | 3.0T, transverse slices parallel to AC-PC line. | SPM 8, P<0.01 threshold 10 contiguous voxels.  Clusters p< 0.05 corrected & uncorrected. | Upregulation of craving all participants food>neutral ↑ activation in the medial/sup frontal gyrus, ant insula, cuneus, middle occipital, sup parietal, thalamus, caudate and↓ activation in sup parietal and precuneus and temporal cortex. Downregulation of craving food>non-food ↑ activity dlPFC, inf frontal gyrus, ant insula, sup parietal cortex and ↓ activation in the precuneus, post insula, occipital lobe. Downregulation of craving more successful weight loss>less successful weight loss ↑ activation in dlPFC. | Individuals with↑ DLPFC activation when told to resist craving are more likely to have successful weight loss outcomes | Small sample size, predominantly female |
| **McCaffery 2009^50^** |  | 3.0T; transverse plane | AFNI  P<0.001, cluster threshold 100mm^3^ | HW > OB ↑ activation to high energy cues in in sup frontal, precuneus and sup parietal regions. SWL > OB ↑ activation to high-cal cues in in prefrontal regions, occipital, lingual, and fusiform regions and precentral and postcentral gyri. OB >SWL ↑ activation to high-cal cues ant cingulate. | SWL show greater inhibitory brain control and attention in response to food cues. | Self-selected sample, small sample, no indication of method or reason of weight loss. |
| **Tregellas 2011^51^** | Hunger ratings;  Appetite ratings | 3.0T; oblique transverse plane (parallel to the planum sphenoidal) | SPM5  Voxel wise threshold p<0.01 FDR | SWL> HW ↑ activation following eucaloric diet in post cingulate gyrus, lateral inf parietal cortex. Overfed diet > eucaloric state ↑ activation in post cingulate in all individuals, driven by HW. | ↑ in default network activity in SWL compared to HW in eucaloric state. | Small sample, compliance to diet not assessed |
| **Bruce 2011 ^52^** | Anthropometrics; Eating Inventory; Depression Scale | 3.0T, Oblique transverse plane at 40 degree angle | BrainVoyagerQX , ROI and whole brain, ROI P<0.001 uncorrected, min cluster size 3 contiguous voxels, whole brain P<0.05 FDR corrected. | Post-surgery pre-meal Food> non-food ↑ middle frontal gyrus, sup frontal gyrus and ↓ activation in the medial frontal gyrus. Post-surgery post- meal food vs non-food ↓ activation in medial frontal gyrus, insula/operculum, inf frontal gyri, parahippocampal gyrus. Pre-surgery, greater BMI associated with less food vs non-food activation in right middle frontal gyrus pre-meal (r=-0.76 P<0.05). | Changes in brain activation post-surgery consistent with lower levels of food motivation and reward. | Small sample size, no control group |
| **Bruce 2014 ^53^** | Hunger ratings | 3.0T, Oblique transverse plane at 40 degree angle | Brain Voyager QX, cluster threshold P<0.01 min k=7 contiguous voxels, alpha=0.05 corrected | Changes pre- to post- intervention Pre-meal behavioural> bariatric food> non-food ↑ activation in medial prefrontal cortex, precuneus. Post-meal bariatric> behavioural ↑ activity middle temporal gyrus, inf temporal gyrus/occipital. | Method of weight loss (Surgical or behavioural diet) display different changes in brain activation | Small sample, some baseline differences between groups |
| **Weygant 2013 ^54^** | Anthropometrics | 1.5T, transverse plane, parallel to AC-PC line | SPM8, ROI, FDR P<0.025 | Correlations between weight loss and activation in the vmPFC (r=0.86 P<0.0001), ant insula (r=-0.82 p<0.0001 and r=-0.85 P<0.0001), dmPFC (r=0.87 p<0.0001 and r=0.85 P<0.0001). Connectivity of vmPFC with dlPFC, and vmPFC with the dorsal striatum and dmPFC predicted weight loss success. | Behavioural impulse control and neural substrates pre-diet can predict degree of weight loss in obese subjects | Small sample size, no long term follow up data. |
| **Murdaugh 2012**^55^ | Weight loss; Hunger ratings | 3.0T; transverse plane | SPM8  FDR≤0.05, p≤0.01, FWE≤0.05 | OB ↓ activation medial PFC, inf parietal lobule, precuneus, PCC, premotor cortex and angular gyrus post- EatRight intervention. Within the OB group, a positive correlation between % weight change and activation of NAcc, ACC, frontal operculum and insula. Positive correlation 9 month weight maintenance and brain activation post- EatRight in putamen, hippocampus and insula. | ↑ reward related activation at start of weight loss program predicted poorer weight control. | Not imaged in same menstrual phase, intervention compliance not measured. |
| **Nock 2012^56^** | Food image preference rating;  Hunger ratings | 3.0T; transverse plane, parallel to AC-PC line | BrainVoyager QX v2.3.1  Uncorrected p <0.005,  FWE p<0.05 | ↓ activation following intervention in high-cal vs non-food images in the sup frontal gyrus, lateral globus pallidus, cingulate gyrus, thalamus, post cingulate, precuneus, and claustrum. ↓ activation following intervention to low-calorie vs non-food images in the insula, precentral gyrus and middle temporal gyrus. ↑ activation following intervention to high vs low-calorie food in the sup temporal and frontal gyri. Positive correlation %weight change and activation of the OFC (r=0.69, P<0.05) and medial frontal gyrus (r=0.66, P<0.05). | ↑ activation at baseline was associated with less successful weight loss. | Lack of control group, small sample size. |
| **Ochner 2012** ^57^ |  | 1.5T transverse plane parallel to AC-PC line. | SPM5, activation threshold P<0.005 uncorrected, cluster threshold 145 contiguous voxels P<0.05 corrected multiple comparisons whole brain | Changes in activity Fasted Pre vs post-surgery ↓ in activation in sup temporal gyrus, middle temporal gyrus, culmen, middle frontal gyrus, precentral gyrus, sup frontal gyrus, precuneus, declive, insula, claustrum. Presurgery greater activation in fasted vs fed in the precuneus and sup parietal lobules. | Greater responsivity to food cues pre compared to post- surgery | Small sample size, female only |
| **Ochner, 2012**^58^ | Fullness ratings;  Ratings of food desire;  Liking ratings | 1.5T; transverse plane, parallel to AC-PC line | SPM5  Uncorrected p<0.005, cluster extent threshold 152 contiguous voxels. Corrected p<0.05 | Pre->post-surgery: reduction in activation to high> low cal foods in reward related responses in the mesolimbic pathway particularly the lentiform nucleus, putamen, and middle and sup frontal gyri. | Reductions in reward related activation were apparent post-surgery in response to high cal foods | Female only, small sample, no control group, responses of visual and auditory cues combined, short follow up. |
| **Ochner, 2011** ^59^ | Eating behaviours and attitudes questionnaire;  Hunger ratings | 1.5T; transverse plane, parallel to AC-PC line | SPM8  Cluster size threshold 135 contiguous voxels. Uncorrected P<0.005, corrected P<0.05 | High > low cal food ↓ activity post-surgery in VTA, ventral striatum, putamen, post cingulate, dmPFC and lentiform nucleus. Food> non-food ↓ activation post-surgery in response in corticolimbic (PFC) and mesolimbic reward pathway. | Mesolimbic pathway activation ↓ in response to high relative to low-cal foods post-surgery. | Females only, small sample, no control group, responses of visual and auditory cues combined, short follow up. |
| **Healthy weight** | | | | | | |
| **Benedict 2012 ^60^** | BGL; Hunger ratings; Image ratings | 3.0T, transverse plane | SPM8, ROI , P<0.05 FWE | Sleep deprivation> sleep ↑ activity in ACC to food images. Sleep deprived responses in the ACC positively correlated with appetite ratings (r=0.67, p=0.02) | Sleep deprivation associated with ↑ activation of ACC and ↑ anticipation for food | Males only, small sample size |
| **Karra 2014** ^61^ | Appetite ratings; Ghrelin; Image ratings; Hunger ratings | 3.0T, parallel to AC-PC line | SPM8, whole brain correction P<0.05 FWE. Cluster voxel threshold P<0.001 uncorrected. ROI P<0.05 corrected | Fasted AA vs TT↓ activation to food images in the hypothalamus, VTA, substantia nigra, Post insula, globus pallidus, thalamus, and hippocampus. Interaction between fasting state, incentive value of food and genotype in the ant insula, OFC and putamen. | TT and AA genotypes display different responsivity to food images, which may be modulated by ghrelin | Male only, small sample |
| **Kroemer 2012 2013 ^62,63^** | Prospective food consumption; Neuroendocrine analysis | 3.0T, transverse plane, parallel to AC-PC line. | SPM5, Whole brain, uncorrected P<0.001 min cluster size k=20. ROI FWE corrected. | Fasting food>non-food ghrelin correlated with activation in the middle and sup occipital/temporal gyrus, fusiform gyrus, caudate, pallidum, midbrain, Rolandic operculum, amygdala, thalamus, ant cingulate gyrus, hypothalamus. Post- glucose food>non-food ↓ activation to in basal ganglia, medial frontal gyrus, middle temporal gyrus, ACC. Post- glucose food>non-food ↑ activation in the ant precuneus and occipital regions. | Fasting levels of ghrelin modulates activation of brain areas associated with reward, visual processing, taste | Small sample size, no control drink |
| **Evero, 2012^64^** | Appetite questionnaire;  24h dietary recall | 1.5T; transverse plane | SPM8 Whole brain and ROI. Uncorrected p<0.005, Bonferroni correction p<0.05. 5 contiguous voxels | Exercise ↓ activation to high-cal foods vs control in the occipital gyrus, inf frontal gyrus, sup parietal gyrus, putamen and insula and ↑ activation in the precuneus. Exercise ↓ activation to low-calorie foods vs control in the occipital gyrus, inf OFC, sup frontal gyrus and precentral gyrus. No-exercise ↑ activation in the lingual gyrus and ↓ response in the occipital gyrus to high-cal foods vs control. | Exercise ↓ neuronal responses to visual food cues in food reward, and visual attention regions | Small sample size. |
| **Born, 2011^65^** | Hunger and satiety ratings;  Liking and wanting ratings | 3.0T. | BrainVoyager QX ROI v2.1.  FDR q<0.05, Voxel cluster threshold n=4x27mm^3^ | Dietary restraint predicted "liking" task-related signalling in the amygdala, striatum, thalamus, and cingulate cortex. Pre-meal "liking" and "wanting" task-related signalling and pre-meal to post-meal "liking" task-related signalling changes in the NAcc correlated positively with dietary restraint. BMI and hunger predicted "wanting" task-related signalling in the hypothalamus and striatum. Post-meal "liking" task-related signalling in the striatum, ant insula, and cingulate cortex and "wanting" task-related signalling in the striatum predicted energy intake. | Potential 'reward deficiency' which was most apparent in the satiated condition. | Female only, small sample size |
| **Born 2010^66^** | Neuroendocrine analysis  Hunger and satiety ratings;  Liking and wanting ratings | 3.0T. | BrainVoyager QX v2.1. Whole brain and ROI.  FDR P<0.05. Voxel cluster threshold 4x27mm^3^. | ↓Reward related activation in the satiated vs fasting condition. Stress sig. ↓ activation in the OFC, frontal cortex, putamen, amygdala, hippocampus and cingulate cortex while fasted compared to rest condition. ↓ activation of the putamen in the stress condition when satiated. | Stress ↓ sensitivity of the reward system and interferes with food preferences and energy intake. | Female only, small sample size. |
| **Cornier 2010**^67^ | 3 day food diary;  TFEQ;  PFS;  Hunger and prospective consumption ratings | 3.0T; transverse plane, parallel to planum spheniodale | SPM5  Whole brain and ROI  P<0.05 corrected 130 voxels. | Female> male ↑ activation in lateral and dlPFC and parietal cortex. Female> male highly hedonic > neutral food↑ activation of parietal cortex and lateral PFC in response to highly hedonic vs neutral hedonic foods. dlPFC activation to highly hedonic food images negatively correlated with ad libitum energy (r= -0.53, P<0.001) | Gender affects neural responses to food with females displaying greater activation of areas associated with cognitive processing | BMI not just in HW category |
| **Frank 2010**^68^ | Hunger ratings | 3.0T; transverse plane | SPM5 Whole brain  FDR P<0.05, min cluster size 10 contiguous voxels. P<0.001 uncorrected | High cal> low cal ↑ activation in the orbitofrontal lobe, occipital lobe, insula, post cingulum, postcentral lobe, thalamus, sup frontal lobe, medial frontal lobe, ant cingulum in females only. Fasted>satiated ↑activation to high-cal foods in the sup medial frontal lobe, fusiform gyrus, ant fusiform, ant cingulum, middle frontal lobe, olfactory, and fusiform gyrus in females only. | Gender diffs and calorie content of food pictures modulates reward related activation. | Small sample size. |
| **Goldstone 2009^69^** | Food diary;  Breakfast caloric analysis;  Appetite ratings;  PANAS | 3.0T; transverse plane, -30 degree to AC-PC line | SPM5 ROI  P<0.001 uncorrected and cluster >5 contiguous voxels. FDR and FWE P<0.05 | Fasting (skipping breakfast) ↑ activation high-cal > low-cal foods in VS, amygdala, insula, medial OFC, lateral OFC. | Skipping breakfast enhances engagement of brain reward areas to high cal foods | Small sample, breakfast meal not standardised |
| **Coletta 2009^70^** | Herman and Polivy Restraint Scale;  Subjective hunger | 1.5T; transverse plane | SPM5 Whole brain  P<0.001 cluster size threshold uncorrected  Min cluster size 8 contiguous voxels | Unrestrained eaters ↑activation when fasted in the sup temporal gyrus, parahippocampal gyrus, putamen, middle frontal gyrus and when satiated ↑ activation cingulate gyrus, inf frontal gyrus, precuneus, and parahippocampal gyrus. Restrained eaters ↑ activation cerebellum when fasted and ↑activation cerebellum, middle frontal gyrus, sup frontal gyrus, insula, middle frontal gyrus and precentral gyrus when satiated. | ↑ activation of hunger and reward related regions when satiated in Restrained vs Unrestrained eaters. | Female only, small sample size, no control for menstrual cycle, did not investigate whether activation was excitatory or inhibitory |
| **Siep 2009^71^** | Hunger ratings | 3.0T; oblique transverse plane (30^0^ tip) | BrainVoyager QX (v 1.9) Whole brain and ROI  P<0.05 uncorrected, cluster size threshold 10 contiguous voxels | Activity in the lateral and medial OFC, cingulate cortex, caudate putamen, insula and fusiform gyrus stronger for low calorie foods when satiated and stronger for high cal foods when fasted. | Satiated participants showed ↑ responses to low-cal foods while hungry participants showed responses to high-cal foods. | Female only, small sample size |
| **Fuhrer 2008^72^** | Hunger ratings | 3.0T; transverse, parallel to AC-PC line | LIPSIA Whole brain  P<0.001  Min 8 contiguous voxels (>216mm3).  P<0.05 image wise false positive rate. | Fasted> satiated ↑ activation in striate, extrastriate cortex, ant lateral OFC and OFC . Satiated>fasted ↑ activation post middle temporal gyrus. Food> non-food ↑activation in insulae, striate and extrastriate cortex, ant mid PFC, thalamus, and cerebellum. Non-food > food ↑ activity in parietal lobe, mid temporal gyrus. | Brain activation differs based on state of satiety and food vs non-food images | Small sample size, males only |
| **Cornier 2007^73^** | TFEQ;  PFS;  Energy needs;  Hunger and prospective consumption ratings | 3.0T; transverse plane, parallel to planum spheniodale | SPM2 Whole brain and ROI  FDR P<0.05 | Eucaloric state highly hedonic food> non-food ↑ activation inf visual cortices, hypothalamus, PFC, parietal cortex and hippocampus. Neutral hedonic> non-food↑ activation of in insula and dlPFC. Overfeeding state high hedonic ↓ activation hypothalamus. | Higher salience of foods ↑s the attention towards these cues. | Small sample |
| **Uher 2006** ^74^ | 24h food and fluid intake record;  Hunger ratings;  Hormone analysis | 1.5T; transverse plane, parallel to AC-PC line | Whole brain Cluster wise p value < 0.005-0.001  Cluster wise P<0.01 | Food> non-food ↑ activation in fusiform gyrus, lingual gyrus, ant gyrus and insula. Fasted> satiated ↑ activation in fusiform gyrus. Female> male ↑ activation in fusiform gyrus. | Processing of food stimuli is mediated by motivational states and gender. | No food vs non-food contrast, non-standardised meal prior to scan, small sample size |
| **Demos 2011**^75^ | Liking ratings;  Hunger ratings | 3.0T; transverse plane | SPM2 Whole brain and ROI  FDR P<0.05. min threshold 5 contiguous voxels | Dieters> non dieters ↑ activation to food in the middle temporal gyrus, inf frontal gyrus, lateral parietal sulcus, sup frontal gyrus and insula. Non dieters>dieters ↑ activation to food in non-dieters in the inf occipital gyrus and middle frontal gyrus. Milkshake> water preload ↑ activity in the OFC, cuneus and ventral ant cingulate. Water> milkshake preload ↑ activation in the precentral gyrus, middle temporal gyrus, lateral OFC precentral gyrus, and dorsal ant cingulate. | Diet and self-regulatory failures may be mediated by hyperactive reward system and motivational responses to food | Female only |
| **Rolls 2007^76^** | Chocolate ratings;  Chocolate cravers questionnaire | 3.0T. coronal plane | SPM2 Whole brain and ROI  FDR P<0.05, P<0.005 uncorrected | Cravers> non cravers ↑ activation of mid and medial OFC and VS to chocolate image. Cravers > non cravers ↑ activation to dark vs white choc in ACC. | Brain processing of craved foods differs cravers and non-cravers. | Small sample, female only. |
| **Grabenhorst 2013 ^77^** | Food ratings;  PPI brain connectivity | 7.0T, coronal slices | SPM5, whole brain correction P<0.05 FWE at the cluster level, cluster threshold 90-163 voxels. ROI P<0.05 FWE | Subjects rated foods as more pleasant and more attractive when exposed to taste labels compared with health labels. Expected pleasantness and attractiveness ratings were strongly correlated. Pleasantness ratings were higher in the taste label condition compared without labels, Health cost ratings did not differ significantly between label conditions. | Label based marketing can alter neural responses to food and affect food choices | Small sample |
| **Hollman 2012^78^** | Cognitive restraint questionnaire;  Disinhibition of eating questionnaire | 3.0T; transverse plane, parallel to AC-PC line | SPM5 Whole brain  FDR corrected P<0.05 | Sig. ↑ in Regulation of appetite > No Regulation of appetite condition in lateral OFC, inf frontal gyrus, insula, supplementary motor area, and temporo-parietal junction. Sig. positive correlations between cognitive restraint and activation of dlPFC and caudate head in response to tasty food. No sig differences in activation patterns between HW and overweight. | Cognitive strategies can regulate desire for tasty foods. | Small sample size, female only, overweight under represented |
| **Lawrence 2012^79^** | General Food Craving Questionnaire;  Hunger and appetite ratings;  Amount eaten | 3.0T, transverse plane parallel to AC-PC line | FMRIB ROI analysis, whole brain  P<0.05, non-adjusted Spearman’s correlation | Activation of the NAcc to food > non-food cues sig. correlated with chip consumption (r=0.47, p=0.019). VmPFC activation correlated with craving (r=0.45, p=0.025). Low self-control interacted with NAcc activation to predict BMI (p=0.0031). | NAcc responses to food cues predict food behaviours and BMI. | Female only, small sample size, BMI ranges across more than just HW category. |
| **Siep 2012**^80^ | Hunger ratings;  Food cravings | 3.0T; oblique transverse plane (30^0^ tip) | BrainVoyager QX (v 1.9) Whole brain and ROI  P<0.01 uncorrected, P<0.05 corrected, min cluster size 9 contiguous voxels | Upregulation >suppression of appetite↑ activity in the VTA, VS, operculum, post insular gyrus, medial OFC, and vmPFC. Suppression of appetite ↓ activity in the VTA and VS more effectively than cognitive reappraisal. Suppression> cognitive reappraisal of appetite ↑ activation in the ant PFC, L lateral OFC, and dlPFC. | Short term cognitive control strategies can modulate activity within the mesocorticolimbic circuitry. | Female only, small sample, short duration of cognitive strategies, no craving ratings in passive viewing |
| **Gearhardt 2011^29^** | Yale Food Addiction Scale (YFAS) | 3.0T; transverse plane, parallel to AC-PC line | SPM5  FDR P<0.05 Whole brain, P<0.001 uncorrected. Min threshold 3 contiguous voxels. | Food addiction scores correlated with activation in ACC, medial OFC, amygdala. Participants with a high FA > Low FA ↑ activation in dlPFC and caudate. | Relationship exists between indicators of addictive eating (YFAS) and neural activation. | Female only, FWE participants met the criteria FA diagnosis, small sample size; not scanned in same menstrual phase |
| **Schur 2009^81^** |  | 3.0T; transverse plane | FSL v3.3 ROI analysis  P<0.05 corrected | Fattening food>object ↑ activation in the brainstem, hypothalamus, amygdala, PFC, insula, striatum (putamen, caudate), thalamus, and the occipital lobe. Non-fattening food> object ↑ activation in the occipital lobe. High cal>low cal ↑ activation in the brainstem, hypothalamus, amygdala, inf frontal gyrus, insula, striatum (caudate, NAcc, putamen), and thalamus. | Brain regions involved in reward and cognitive processing are selectively responsive to food | Small sample size, female only, no control of menstrual phase, handedness not controlled; BMI not solely in the HW range |
| **Passamonti 2009^82^** | DEBQ (external food sensitivity);  Hunger ratings;  PPI brain connectivity | 3.0T; coronal plane | SPM5 ROI  ROIs FWE P<0.05  Whole brain P<0.001, 10 contiguous voxels | Appetising>bland foods ↑ activation in VS, amygdala and ACC. Changes in connectivity between the frontal pole, post parietal cortex and VS evident to appetising vs bland foods, and motivated by external food sensitivity. | Individuals with high external food sensitivity have altered brain responses to appetising food | Small sample size, BMI across multiple categories |
| **Killgore 2003, 2005, 2006 ^83-85^** | 24h recall;  Motivational salience of stimuli; PANAS | 1.5T; coronal plane | SPM99 Whole brain and ROI  P<0.005 uncorrected, min 10-20 contiguous voxels, p<0.05 corrected | High cal> low cal↑ activation in the medial and dlPFC, thalamus, cerebellum, medulla, and the middle occipital gyrus. Low cal> high cal ↑ activation of the middle temporal gyrus, lingual gyrus and medial frontal gyrus. BMI negatively correlated with activation of inf orbitofrontal gyri and ant cingulate gyrus to high-cal foods (P<0.001) and activation of the inf orbitofrontal gyrus to low-cal foods (P<0.001). Negative affect associated with ↑ activation in OFC, ant cingulate gyrus and post insula when viewing high cal foods. | High cal food ↑ activation in regions processing motivational salience of food stimuli compared to low cal food | BMI range not solely in the HW category, not scanned in same menstrual phase, female only, small sample. |
| **Porubska 2006^86^** | Appetite ratings | 1.5T; transverse plane | SPM2 ROI and whole brain  P<0.05 FDR | Food> non-food ↑ activation of the OFC and insula/opercular cortex. | Brain activation modulated by the subjective feeling of appetite. | Small sample size |
| **Simmons 2005^87^** |  | 3.0T; transverse plane, ROI | SPM99 Whole brain  P<0.005 uncorrected, min 7 contiguous voxels, P<0.05 uncorrected | Food >non-food ↑ activation in the insula/operculum, OFC, ACC, inf occipital gyrus, inf temporal gyrus and fusiform gyrus. | Gustatory cortices associated with taste and reward are activated in response to food pictures, not just actual food. | Small sample size, males only |
| **Overweight/obese** | | | | | | |
| **Geliebter 2013 ^88^** | Anthropometrics; Eating Disorder examination; Hunger and fullness ratings | 1.5T, transverse plane | SPM8, whole brain P<0.005 uncorrected, cluster threshold 50 contiguous voxels. Contiguous clusters k>147 sig at P<0.05 corrected. | Fasted male>female high>low cal ↑ activation inf parietal lobule. Fasted female>male high>low cal cues ↑ activation caudate nucleus and temporal occipital area. Satiated male>female high>low cal ↑ activation in the fusiform gyrus. Satiated female>male high> low cal ↑ activation in dorsal ACC. | Fasted females had ↑ activation in affective and reward related processing regions, satiated had ↑ activation in control regions. | No control for menstrual cycle, images not matched, no control group |
| **Lundgren 2013 ^89^** | Anthropometrics; Three day recall; Percent energy consumed after evening meal | 3.0T, oblique transverse plane angle of AC-PC line 17-22 degrees | Brain Voyager QX, whole brain, P<0.05 whole brain corrected | Control> EH pre-meal food> blurred images ↑ activation inf parietal lobule, precentral gyrus, cingulate gyrus. Post- meal EH>Control food>blurred ↑ activation fusiform gyrus. Control>EH food>blurred inf frontal gyrus, inf parietal lobule. Pre-meal food>animal sig interaction in inf frontal gyrus, sup temporal gyrus, middle temporal gyrus, cerebellum. EH ↓ activation pre- to post- meal for food images. | People with EH are less reactive to food cues in brain areas associated with sensorimotor and cognitive attention and processing when fasted | Small sample size, no HW controls, readings taken during the day rather than at night |
| **Tryon 2013** ^90^ | Stress Inventory; Food consumption; Saliva; Functional connectivity, Cortisol concentration | 3.0T, sagittal slices | SPM8, ROI p<0.01, FDR P<0.05 | High stress> low stress high cal >non-food ↑activation ACC, amygdala, medial OFC, putamen and caudate. High stress vs low stress non-food> high cal ↑ activation in the caudate and putamen, ant PFC, ant cingulate, dlPFC, lateral OFC. High stress>low stress low cal>control ↑ activation in the caudate. High stress> low stress High cal> low cal ↑ activation in the amygdala, ant cingulate, med OFC, putamen, caudate. High stress > low stress low cal>high cal ant PFC, dlPFC. | Chronic stress may affect eating habits and quantities eaten, leading to overeating. | Sig difference in age, female only |
| **Van Vugt 2012 ^91^** | OGTT (insulin sensitivity); BGL; Questionnaire for hunger and wellbeing | 3.0T, transverse plane | SPM8, ROI, P<0.001 uncorrected, clusters >4 contiguous voxels P<0.05 FWE | High cal food> control all subjects ↑ activation in dlPFC, mPFC, insula, NAcc, ventral pallidum, putamen, amygdala, caudate, VTA, substantia nigra, hippocampus, pulvinar and midbrain. All subjects low cal food>control ↑ activation dlPFC, insula, amygdala, hippocampus, pulvinar, midbrain. Activation to high cal food in the dlPFC, ant cingulate, midbrain negatively correlated with insulin sensitivity. High cal-low cal contrast activation in the OFC, dl PFC, insula, ventral pallidum VTA and midbrain positively correlated with insulin sensitivity. | Insulin sensitivity is an important determinant of brain responsiveness to food cues. | Small sample size |
| **Luo 2012** ^92^ | Anthropometrics; Hunger and desire to eat ratings | 3.0T | FMRIB P<0.05 corrected for whole brain comparisons, ROI | Food>non-food ↑ activation OFC, ventral medial PFC, ACC, insula, nucleus accumbens, amygdala, hippocampus and occipital cortex. High cal> non-food ↑ activation insula, OFC, lateral PFC, ACC amygdala, striatum. High cal>low cal ↑ activation ACC, medial PFC, OFC, hippocampus, insula, lateral occipital cortex. Brain responses to high cal foods in OFC, striatum, insula, amygdala mPFC positively correlated with waist circumference (r=0.62, p=0.03). | High cal foods activate reward and motivation related brain regions and ↑ levels of abdominal fat predict brain reward activity. | Small sample size, not all in obese category, abdominal fat not directly measured. |

^a^ Visual cue classification has used the authors description where possible^, b^ Only results from OB and HW populations reported

ACC= anterior cingulate cortex, ant= anterior, BMI= body mass index, Cal=calorie, DBEQ=Dutch Eating Behaviour Questionnaire, diff = difference, FDR= false discovery rate, dl= dorsolateral, FFA= free fatty acids, FEW= family wise error, HW= healthy weight, NAcc= nucleus accumbens, OB= obese, OFC= orbitofrontal cortex, OW= overweight, PANAS= Positive and Negative Affect Scale, PFC= prefrontal cortex, post=posterior, ROI= region of interest, RYGB= Roux-en-Y gastric bypass, sig = significantly, sup=superior, SWL= OB participants who had successfully lost weight, TFEQ= Three Factor eating Questionnaire, VS= ventral striatum, vs= versus, VTA= ventral tegmental area, YFAS=Yale Food Addiction Scale, PFS= power of food scale, PPI= Psychophysiological interaction, vm= ventromedial, VS= ventral striatum, VTA= ventral tegmental area.
