# Supplementary material for: Neural Responses to Visual Food Cues According to Weight Status: A Systematic Review of Functional Magnetic Resonance Imaging Studies
Source: Front Nutr. 2014 Jul 9;1:7. doi: 10.3389/fnut.2014.00007 (PMC4428493; doi:10.3389/fnut.2014.00007)
Supplement: Supplementary file 4 [file Data_Sheet_4.DOCX]

**Table 4:** Locations of clusters (MNI) with significant ALE values for studies reporting decreases in activation from pre- to post- weight loss included in the meta-analysis.

| **Cluster^a^** | **Anatomical label^b^** | **Peak voxel coordinates^c^** | | | **Cluster size (mm^3^)** | **ALE value (x10^-3^)** |
| --- | --- | --- | --- | --- | --- | --- |
|  |  | x | y | z |  |  |
| 1 | L Superior temporal gyrus | -40 | -48 | 6 | 256 | 8.4 |
| 2 | R Middle frontal gyrus | 32 | 34 | 34 | 216 | 7.6 |
| 3 | L Lentiform nucleus | -12 | 0 | -2 | 192 | 7.8 |
| 4 | L Cingulate gyrus, Brodmann area 23 | -4 | -34 | 26 | 176 | 8.7 |
| 5 | R Precentral gyrus | 40 | 0 | 42 | 160 | 8.6 |
| 6 | L Cingulate gyrus, Brodmann area 32 | -6 | 24 | 42 | 152 | 8.6 |
| 7 | R Cingulate gyrus, Broadmann area 31 | 4 | -36 | 38 | 144 | 8.5 |
| 8 | R Precuneus, Brodmann area 7 | 16 | -72 | 40 | 144 | 8.6 |
| 9 | R Cingulate gyrus, Brodmann area 32 | 2 | 16 | 42 | 144 | 8.5 |
| 10 | R Inferior parietal lobule | 50 | -40 | 38 | 136 | 8.4 |
| 11 | R Precuneus, Brodmann area 7 | 20 | -52 | 44 | 136 | 8.4 |
| 12 | R Superior occipital gyrus | 36 | -74 | 26 | 128 | 8.4 |
| 13 | L Middle temporal gyrus | -28 | -64 | 22 | 120 | 7.3 |

^a^ Clusters surviving FDR threshold P=0.05, ^b^ L= left, R=right, ^c^ Coordinates reported in standardised Montreal Neurological Institute coordinates
